# XGBoost-Based Digital Twin Model for Predicting Trajectory Errors in a Hexapod Coordinated Machining System Using Positioning Accuracy and Vibration Data

**DOI:** 10.3390/s25237142

**Published:** 2025-11-22

**Authors:** Kanglin Xing, Miao Feng, Ilian A. Bonev, Henri Champliaud, Mohamed Cheriet, Zhaoheng Liu

**Affiliations:** 1 Department of Mechanical Engineering, École de Technologie Supérieure, 1100 Notre-Dame St W, Montreal, QC H3C 1K3, Canada; kanglin.xing@etsmtl.ca (K.X.); henri.champliaud@etsmtl.ca (H.C.); 2 Department of Systems Engineering, École de Technologie Supérieure, 1100 Notre-Dame St W, Montreal, QC H3C 1K3, Canada; miao.feng@etsmtl.ca (M.F.); ilian.bonev@etsmtl.ca (I.A.B.); mohamed.cheriet@etsmtl.ca (M.C.)

**Keywords:** digital twin, trajectory errors, XGBoost, hexapod coordinated machining system, vibration, positioning accuracy

## Abstract

Dynamic errors in robotic machining can degrade part quality, particularly in flexible platforms that are susceptible to both geometric and inertial disturbances. This work introduces a data-driven digital twin for pointwise prediction of circular trajectory errors in a hexapod-based machining cell, using a compact sensing configuration that combines ballbar measurements with tri-axial vibration signals. Deviations measured by ballbar, acceleration data, and CMM-measured profiles are synchronized in the angular domain via a unified pipeline for denoising, resampling, and phase alignment. Sliding-window vibration statistics and the ballbar path error are used as inputs to XGBoost, multilayer perceptron, and random forest regressors. Model performance is evaluated under a deployment-relevant leave-one-run-out protocol and a conventional random 70:30 point split. XGBoost achieves micrometer-level accuracy on unseen runs, with RMSE around 5 µm, R^2^ exceeding 0.80, and near-complete coverage within a ±20 µm tolerance band. Compared to baseline models, it also provides improved suppression of extreme residuals. Feature importance and ablation studies show that the ballbar path error captures the dominant geometric component, while compact hybrid feature sets—combining this anchor with selected vibration descriptors—retain most of the predictive accuracy and enable practical offline batch-level compensation.

## 1. Introduction

Robotic circular-path machining requires accurate prediction and compensation of path errors, subject to practical constraints related to cost, space, and integration [1]. Circular-path features are common in rings, bores, and coupling interfaces, where profile accuracy governs assembly and sealing. Curvature amplifies compliance deflection, tool runout maps to periodic radius error, interpolation and corner smoothing introduce bias, and chatter or stick–slip appears as localized radial peaks [2]. Limited fixture stiffness and workspace-dependent robot rigidity couple in-plane and out-of-plane deviations, so single-modality sensing often fails to capture the full error picture [3,4]. Predicting circular-feature quality is valuable. It enables pre-emptive path-error compensation that reduces scrap and rework in cost- and space-constrained cells. It supports adaptive feeds and batch reuse of compensation to improve throughput and consistency. It also localizes disturbance-prone arc segments for targeted process tuning without resorting to expensive 3D metrology [5].

Circular-path error is commonly measured using laser trackers, ballbar systems, optical motion capture system, and on-machine probing, each presenting trade-offs between observability and deployability [6]. Beyond standard no-load DBB tests, a Loaded Double Ball Bar (LDBB) device that enables circular testing of machine tools under controllable cutting-like loads has been proposed [7]. Full 3D metrology using laser trackers enables high-fidelity trajectory reconstruction; however, line-of-sight requirements, floor-space limitations, and cost considerations restrict deployability in compact manufacturing cells [6]. Conventional ballbar instruments are cost-effective and straightforward to deploy, providing direct quantification of in-plane radial deviations. Nevertheless, they offer limited visibility into out-of-plane deviations and process-induced dynamic effects, which often dictate machining quality [8]. Machining quality assessment and geometric-error prediction are typically based on vibration/acceleration, acoustic emission, cutting force or spindle power, servo and encoder signals, on-machine geometric probing or laser-displacement measurements, and vision-based or surface-imaging data [9,10]. Vibration sensing is widely accessible and sensitive to process anomalies, although its correlation with geometric path errors is influenced by structural dynamics and operating conditions [9,10]. These trade-offs underscore a persistent gap between shop-floor feasibility and the level of observability required for reliable path error prediction and compensation [1].

Digital twins for robotic machining have emerged as a means to integrate sensing, modeling, and decision-making [11]. For industrial robots, joint elasticity, workspace-dependent stiffness, and controller latency present challenges to fully online digital twins capable of closing the loop during cutting operations [1]. As a result, many practical implementations adopt a staged approach. Initially, offline twins facilitate data acquisition, trajectory evaluation, and compensation planning under relaxed latency constraints. These systems may later transition to online operation once controller access, synchronization, and timing requirements are fulfilled [12]. This staged paradigm offers an attractive compromise, maintaining practical deployability while enabling a structured pathway toward real-time compensation [13].

This study situates a lightweight digital twin within this staged framework and addresses the observability gap through a cross-modal, data-driven approach. Geometric deviations measured via ballbar instrumentation are fused with statistical features extracted from accelerometer signals. The underlying premise is that in-plane deviations captured by the ballbar encode quasi-static geometric and compliance errors, whereas vibration-derived statistics reflect dynamic disturbances encountered during motion or cutting. By learning a point-wise mapping across these modalities along the circular trajectory, the model is capable of capturing a broader error manifold than either sensing modality alone, while retaining the low cost and ease of deployment associated with ballbar-based setups. The primary contributions of this work are summarized as follows: (1) A cross-modal fusion framework is proposed, integrating ballbar-derived geometric deviations with vibration statistics for point-wise prediction of circular-path errors in robotic machining; (2) A comparative evaluation of XGBoost, random forest (RF), and multilayer perceptron (MLP) models is performed using the complete and reduced feature sets, including an analysis of model robustness with respect to modality-based feature grouping; (3) A lightweight digital twin prototype is implemented to support offline trajectory evaluation and compensation planning, with a clearly articulated pathway for transitioning to online deployment that addresses interface design and latency constraints.

The structure of this paper is as follows. In Section 2, related studies on circular-path assessment method, vibration-informed quality prediction, and data-driven digital twins for robotic machining are reviewed. Section 3 is devoted to the description of the system architecture and data pipeline. In Section 4, signal synchronization, feature engineering, and model design are formalized, with emphasis on baseline selection and hyperparameter tuning. The training protocol and evaluation metrics are outlined in Section 5. Experimental results, including model performance, feature importance, and analyses of generalization and robustness, are presented in Section 6. The LabVIEW-based implementation of the digital twin is detailed in Section 7. Practical implications and limitations are discussed in Section 8. Finally, Section 9 concludes the paper and proposes directions for future work.

## 2. Related Work

Across recent studies, a common pipeline has been established: multi-source signals are acquired, aligned and fused in a unified parameter domain, a digital-twin model is constructed, and the twin is used for quality and geometric-error prediction and compensation. Geometric and external metrology provide traceable scale and reference trajectories—for example, ballbar, laser tracker, and on-machine or optical geometric sampling [6,14,15,16]. Process and controller signals capture dynamic disturbances and machine states, including vibration or acceleration, acoustic emission, cutting force and power, spindle and servo currents, encoder and following error, and vision-based surface cues [17,18,19]. Recent studies have successfully fused such multi-source signals for quality prediction. For example, vibration and acoustic emission data have been combined in hybrid deep learning models to predict surface roughness in precision grinding operations, achieving higher robustness than single-sensor approaches [20]. Other works fuse vibration with cutting force, spindle current, or in-process vision to estimate tool wear, surface roughness, or dimensional deviation under varying cutting conditions [21,22]. Slow-drift variables such as temperature and thermal elongation support long-horizon stability and compensation. A practical rule is that scale is stabilized with a small number of high-trust geometric anchor points, dynamics are characterized with high-rate process signals, and a deployable mapping is then built in a unified path or angle domain. In drilling or milling, real-time laser-tracker loops have demonstrated “measure–estimate–correct” with notable error reduction, ballbar has enabled low-cost and rapid in-plane circular-path assessment well suited as a geometric anchor, and on-machine or optical methods provide periodic verification and calibration when continuous tracking is infeasible [6,14,15].

Alignment and fusion are critical for converting heterogeneous data into learnable samples. Because feed, posture, and tool condition introduce phase shifts, naïve time-axis concatenation can produce spurious correlations. Angle- or arc-length parameterization has therefore been used: high-rate process features such as vibration, power, current, and servo error are resampled onto a common path coordinate and aligned point-wise with geometric labels such as ballbar-interpolated radius deviation, on-machine probe points, or optically reconstructed tool center position pose [17,18,19]. Along this route, surrogate modeling of quality has been validated in turning and milling. Vibration-based GPR, ELM, and tree ensembles have shown strong utility and calibrated uncertainty across materials and cooling regimes, and position-aware monitoring has demonstrated the generalization benefits of path-domain alignment; deep fusion of dynamic process and morphology features has further improved robustness relative to single-channel baselines [17,23].

For digital-twin modeling, physics-based, data-driven, and hybrid routes are commonly combined. Physics-led twins centered on posture-dependent stiffness or chain dynamics are well suited to offline compensation and extrapolation and are typically calibrated or verified with external metrology such as laser trackers or large-volume optical systems [24]. In addition, Denavit–Hartenberg-based kinematic chains and homogeneous transformation matrices are widely used to propagate link and joint errors to the tool center point. For example, a homogeneous-matrix formulation has been used to analyze assembly error propagation in multitasking machines and quantify volumetric accuracy in the working volume [25]. Data-driven twins emphasize low intrusion and rapid deployability, using tree models, kernel methods, or lightweight neural networks for point-level mappings, while leveraging ensemble structures such as boosting for small industrial datasets [26]; multi-sensor fusion frameworks and transformer-style encoders have further enhanced wear and roughness prediction stability [18,27]. However, the flexibility of such advanced learners often comes at the cost of transparency, and their “black-box” nature can limit trust and adoption on the shop floor. To mitigate this, explainable artificial intelligence (XAI) methods are increasingly used to add physical interpretability to data-driven manufacturing models. For example, SHapley Additive exPlanations (SHAP) and related attribution techniques have been applied in an electrochemical machining twin to inspect model decisions in real time, diagnose process anomalies, and ensure that predictions remain consistent with known cavity-formation physics [28]. Motivated by this trend, the present study complements its XGBoost-based digital twin with a global feature-importance and ablation analysis, providing a lightweight form of XAI that clarifies the relative roles of ballbar and vibration inputs.

Closer to the present approach, Yao et al. proposed an ultrasonic–AI hybrid framework in which an enhanced XGBoost model with Bayesian optimization is used to predict void defects in concrete-filled steel tubes [29]. Their results highlight the capability of boosted tree ensembles to capture nonlinear relations between ultrasonic features and structural defects. Hybrid twins connect physics and data through residual learning or online correction and are increasingly viewed as a practical bridge to shop-floor adoption; recent quality-oriented DT reviews also describe a phased trajectory that is offline first and then progressively online once interfaces and timing budgets allow [12,30]. For hexapod-based robotic machining cells, previous work by the authors has experimentally quantified how spindle speed, feed rate, and depth of cut influence dynamic trajectory errors under circular interpolation [31], providing an important empirical basis for the present digital-twin study. For large cells and robotic workspaces, large-volume triangulation and optical motion capture can serve as high-observability calibration channels, although dynamic accuracy, occlusion, and setup cost require algorithmic compensation and careful layout [32,33].

As for the implementation of the DT model in machining quality prediction, artificial neural networks and other machine learning models have been widely adopted. For example, multilayer perceptron and radial basis function networks have been used to predict dimensional error on inclined surfaces manufactured by ball-end milling, achieving micrometer-level accuracy from cutting parameters and surface slope [34]. More broadly, ANN- and ensemble-based schemes have emerged as a strong reference baseline in drilling and milling quality prediction and monitoring [12,30]. Nevertheless, three limitations remain prominent: point-level trajectory errors are rarely modeled explicitly, cross-modal fusion between geometric and vibration measurements is uncommon, and few studies provide digital-twin pipelines that generate deployable error-compensation tables.

Towards these limitations, in this work, a small set of geometric anchor points is used to stabilize scale; process features are aligned in the circular-angle domain; interpretable, compact models are trained to learn point-level error and generate offline compensation; and interfaces are kept ready for migration to online closed loop and virtual commissioning. This data-first path binds quality and geometric error in one domain and balances observability with deployability [6,12,14,30].

## 3. System Architecture and Data Pipeline

### 3.1. Digital Twin Architecture

**Figure 1:**
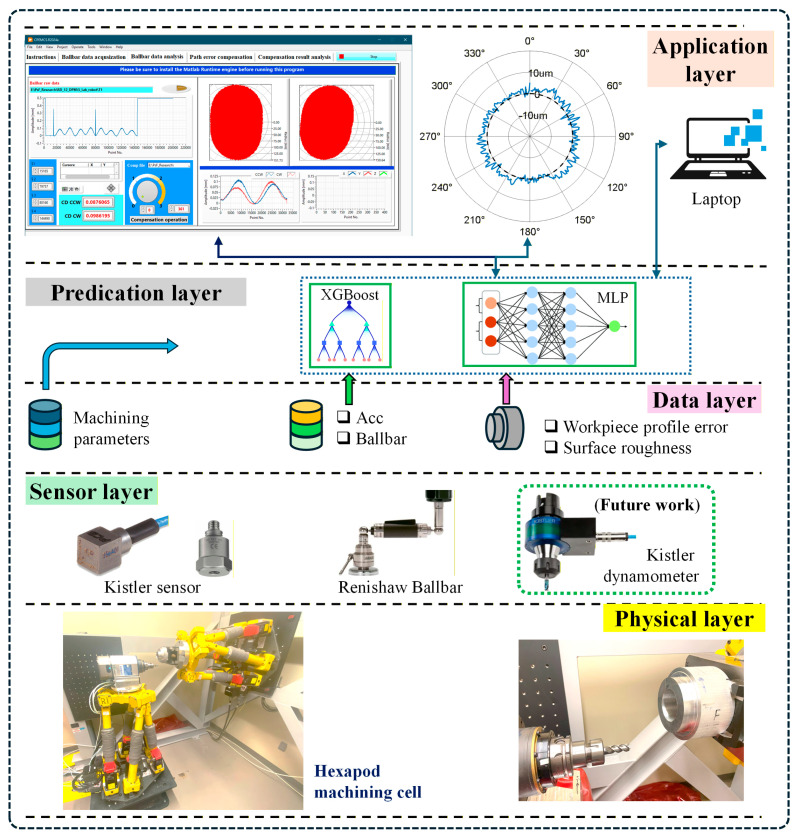
System architecture for the proposed digital twin system. The design omits a traditional physics-based virtual model, since prediction relies entirely on real sensor data and data-driven learning. Data flow is bottom-to-top, while commands flow top-to-bottom. This data-driven framework supports high-resolution, per-point accuracy estimation. The twin mirrors both robot behavior and machining process state to predict trajectory errors and generate trajectory-compensation values.

The proposed digital twin system for hexapod-based robotic machining is designed as a modular, multi-layer architecture that integrates synchronized in-process sensor data acquisition, data processing, machine learning-based prediction, and visual feedback within a unified framework. The architecture is composed of five functional layers: the Physical Layer, Sensor Layer, Data Layer, Prediction Layer, and Application Layer, as illustrated in Figure 1.

Physical Layer: This layer constitutes the physical machining environment where all sensing signals and machining phenomena originate. It includes the dual-hexapod robotic platform (FANUC F-200iB, FANUC, Oshino-mura, Minamitsuru-gun, Yamanashi, Japan), aluminum cylindrical workpieces, the spindle and cutting tool system, as well as all mechanical fixtures used for sensor mounting. In the system configuration, the tool is mounted on one hexapod and the workpiece is rigidly fixed on the other, allowing precise spatial coordination to execute circular machining paths. No real-time compensation is applied during the machining process, and consistent cutting parameters across all experimental runs are used. The physical layer provides the real-world foundation for sensor data collection and model training, directly influencing the fidelity of the digital twin system.

Sensor Layer: This layer consists of hardware components responsible for synchronized in-process data acquisition during machining. It includes triaxial vibration sensors mounted on the spindle and tool platform to capture dynamic responses, and a telescopic ballbar system to measure pre-machining trajectory errors along the circular path. Each experiment captures vibration signals and interpolated radius deviations at 360 angular points.

Data Layer: Raw sensor data is transmitted to this layer through a LabVIEW interface and stored in structured Excel or CSV format. In this layer, data preprocessing including noise filtering, interpolation, and feature extraction are conducted either in LabVIEW (2024, Q1, 64-bit) or through Python scripts (version 3.13.6). The output of this layer is a set of time-aligned, per-point feature vectors that include vibration descriptors (e.g., RMS, mean, kurtosis) and corresponding ballbar errors.

Prediction Layer: This layer hosts the machine learning models used to estimate machining error at each trajectory point. The main model is an XGBoost regressor trained on historical data collected from 19 machining runs. Each input vector corresponds to a unique angular point and includes both vibration features and ballbar measurements. The prediction output is the estimated radius error, trained against ground truth coordinate measuring machine (CMM) data. For comparison, a MLP and random forest (RF) model is chosen as the baseline because of its common success in most domains.

Application Layer: The top layer is responsible for visualizing predicted machining errors and supporting feedback decisions. It is built in LabVIEW and presents trajectory error maps, per-point predictions, and cumulative deviation curves. The application layer enables users to assess machining quality for each run shortly after completion. In this study, compensation is applied offline between runs. In future implementations, the same trajectory-compensation values could be streamed to the controller to enable online compensation along the toolpath via the Dynamic Path Modification (DPM) function.

By connecting these layers, the digital twin enables high-resolution, per-point error prediction and visualization, forming a feedback loop between physical machining and virtual analytics. This architecture primarily supports offline evaluation and batch-level compensation, and provides a basis for future extensions toward online adaptation and real-time compensation strategies.

### 3.2. Data Acquisition Protocol

**Figure 2:**
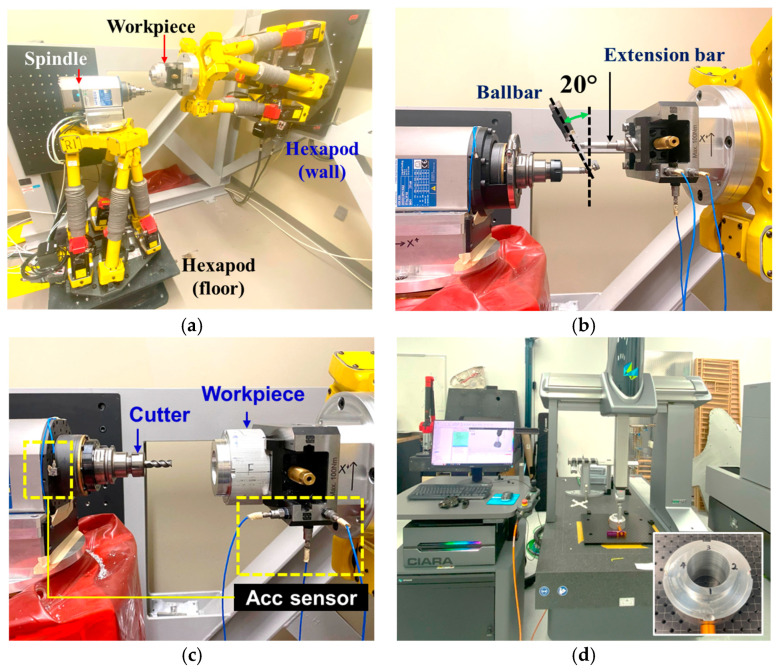
Setups for robotic milling of aluminum cylinder workpiece: (**a**) setup of hexapod machining cell using two FANUC hexapods; (**b**) Installation of ballbar with the length of 50 mm for the measurement of the circle with the radius of 47 mm using small circle adaptor [35]; (**c**) Robotic milling of workpiece with the milling process monitoring using three single axial accelerometers in the X, Y and Z directions; (**d**) Measurement of dimension and circularity of workpiece using CMM.

The experimental campaign was carried out using a hexapod-based robotic machining cell consisting of two FANUC F-200iB hexapods (FANUC, Oshino-mura, Minamitsuru-gun, Yamanashi, Japan) (Figure 2a). The workpiece was fixed on a clamp attached on the wall-mounted hexapod, while the cutting tool was attached to an electric spindle installed on the floor-mounted hexapod. Circular path error measurements were performed using a Renishaw QC20-W telescopic ballbar system (Renishaw, New Mills, Wotton-under-Edge, Gloucestershire, UK), featuring a sampling rate of 1000 Hz, implemented through an API-based interface and a custom-designed small-circle adaptor (Figure 2b). The ballbar sensor provided a measurement accuracy of 0.1 μm under standard conditions at 20 °C, with a measurement range of ±1.0 mm. An API-based data acquisition strategy was employed to record ballbar data at a consistent sampling rate of 1000 Hz. To monitor vibrations during the machining process, three uniaxial accelerometers (Model: 352C04, PCB Piezotronics, Inc., Depew, NY, USA) were mounted on the workpiece side (Figure 2c). These sensors were configured to measure vibration components along the X, Y, and Z axes, with each axis monitored by a dedicated accelerometer. The following parameters were selected for vibration measurement: (1) sampling frequency of 2048 Hz; (2) spectral resolution of 0.125 Hz; and (3) sensor sensitivity of 100 mV/g. Vibration signals were acquired using an LMS SCADAS data acquisition system (Model: SCM01, LMS International n.v., Leuven, Belgium) and were subsequently analyzed in the time domain using LabVIEW.

For consistency, all sub-millimeter dimensional quantities in this paper are reported in micrometers and all temporal sampling rates in Hertz. Before mapping the signals to the angular domain and performing spline-based resampling, the ballbar and acceleration time series are additionally processed with zero-phase low-pass Butterworth filters to remove high-frequency components above the effective analysis bandwidth and to mitigate aliasing, as detailed in Section 4.1.

The machining data were obtained from robotic machining experiments conducted on an aluminum cylindrical workpiece. A 6061-T651 aluminum cylinder (Grainger Canada, Montreal, QC, Canada) with a radius of 55 mm was employed, and a NIAGARA end mill (Seco Tools in USA and Canada, Bellingham Drive, MI, USA), specifically designed for aluminum alloys, with a diameter of 12.7 mm, was utilized (Table 1). Owing to the absence of a coolant system in the hexapod-based machining cell, a small radial depth of cut of 100 µm was selected. Even at a 100 µm radial depth of cut, accelerometer signals remain distinguishable from the background when appropriate band-limiting and feature extraction are applied, consistent with prior reports on low-engagement milling [36]. The remaining cutting parameters were set as follows: a spindle speed of 10,000 RPM, an axial depth of cut of 8 mm, and a feed rate of 60 mm/min. These conditions correspond to a relatively light finishing regime; generalization to heavier roughing cuts, different materials and tooling, or significantly different engagement levels remains to be investigated in future work.

A total of 19 machining trials for aluminum cylinder parts were carried out under fixed cutting parameters, each involving the execution of a circular trajectory with a nominal radius configured via an out-of-plane ballbar adaptor. During each trial, the pre-machining trajectory deviation was recorded by the ballbar system using for path error compensation. Using the updated circular path trajectory program, vibration signals along the X, Y, and Z axes were collected in real time during the machining process. After machining, a Hexagon CMM was used to evaluate the post-machining radius error at 360 uniformly sampled angular positions per trial (Figure 2d). The complete dataset includes synchronized records of interpolated ballbar errors, segmented vibration features, and labeled CMM measurements, forming the input-output basis for per-point error prediction.

To quantify random uncertainty (Type-A), we performed five repeated ballbar measurements in the machining direction (CCW). Using the ring-level approach, each circle was compressed to a root mean square (RMS) of full circle data. The across-run sample standard deviation of RMS divided by five yields the Type-A standard uncertainty (0.17 µm). Details are provided in Appendix A.

## 4. Feature Engineering and Model Development

### 4.1. Signal Synchronization

To enable pointwise comparability across channels with differing native sampling schemes, a unified synchronization pipeline is applied to all runs. This workflow consists of time-domain denoising, angular trajectory construction, resampling on a unified angular grid, direction and phase alignment to the CMM profile, and angle-node feature extraction. The pipeline standardizes data representation, mitigates phase errors and aliasing during resampling, and ensures compatibility with downstream training and evaluation processes based on a common angular index.

(1) Time-domain denoising and stability preprocessing. Ballbar and tri-axial acceleration time series are first subjected to zero-phase, 4th-order Butterworth filtering via the filtfilt function. Cutoff frequencies are fixed at 50 Hz for ballbar signals (sampled at 1000 Hz) and 600 Hz for accelerometer data (sampled at 2048 Hz), effectively attenuating high-frequency noise without introducing phase lag. The CMM contour, defined in the angular domain, is not filtered numerically. To enhance numerical stability during filtering, short gaps and impulsive spikes are repaired using linear interpolation and localized smoothing. Filtering is performed prior to synchronization to suppress aliasing when remapping high-frequency time-domain signals onto a discrete angular grid.
(2) Angular trajectory construction and one-revolution normalization. From the filtered ballbar signal $b_{f}(t)$, the analytic signal is computed and its instantaneous phase ϕ(t) is unwrapped. A normalized angular trajectory θ(t) over a single revolution interval $\left[t_{0},t_{1}\right]$ is then defined as:
  (1) φ(t)=unwrap(arg{hilbert(bf(t)−bf)})
  (2) $$\theta (t)=360{}^{\circ}\cdot \frac{\varphi (t)-\varphi (t_{0})}{\varphi (t_{1})-\varphi (t_{0})}\;mod\;360{}^{\circ}$$
  This procedure yields a strictly monotonic, endpoint-continuous angular trajectory directly inferred from the measured ballbar signal and therefore avoids the limitations of a constant-speed assumption or encoder-free linear time-to-angle mapping.
(3) Resampling on a unified 1° angular grid. The inverse mapping $t(\theta_{k})$ is obtained via linear interpolation of θ(t), where $\theta_{k}\in \{0^{\circ },1^{\circ },\ldots ,359^{\circ }\}$. The filtered ballbar and acceleration signals are resampled at these timestamps to yield uniformly angle-indexed sequences $b(\theta_{k})$ and $x/y/z(\theta_{k})$. Near boundary regions or sparsely sampled segments, linear interpolation and controlled extrapolation are employed to maintain numerical stability and endpoint continuity. The unified 1° angular grid serves as a master index across all sensing channels, thereby facilitating feature construction and model integration.
(4) Direction and phase alignment to the CMM measured workpiece profile. To compensate for arbitrary sensor mounting orientations and initial angle offsets, the zero-mean ballbar signal is aligned with the CMM profile $g(\theta_{k})$ through exhaustive circular shifting over 0–359° and optional signal reversal. The transformation that maximizes the correlation coefficient is selected. The same flip-and-shift operation is applied to the tri-axial acceleration signals to preserve angular coherence across channels. This step ensures geometric alignment between ballbar and CMM data, thereby eliminating systematic angle misalignment during model training.

Finally, this procedure results in a reproducible, angle-indexed dataset suitable for robust model development, cross-validation, and comparative evaluation under the designated protocols.

### 4.2. Feature Extraction

The raw vibration signals collected in the machining process and ballbar results measured before machining were processed in the time domain for feature extraction. Five standard statistical features were included in this work for acc feature extraction: Mean, Standard deviation (SD), Root Mean Square (RMS), Peak-to-Peak Value (P2P) and Crest Factor (Cf) [37] (Equations (3)–(7)). Precisely, Mean represents the average amplitude of the vibration signal, offering a baseline indication of signal offset. SD measures the dispersion of the signal around the mean, capturing the amplitude variability. RMS quantifies the overall energy content of the signal. P2P captures the maximum amplitude range between the signal’s highest and lowest values. Crest factor evaluates the peak amplitude to the RMS value, highlighting extreme transient spikes. Suppose the sample set $X=(x_{1},x_{2},\cdots ,x_{N})$ has the mean is $\bar{x}$, then, the statistical features are formally defined as follows:

(3) $$\mathrm{Mean}\;(\bar{x})\;=\;\frac{1}{N}\sum_{n=1}^{N}x_{n},$$

(4) $$SD=\sqrt{\frac{1}{N}\sum_{n=1}^{N}{\left(x_{n}-\bar{x}\right)}^{2}}$$

(5) $$RMS=\sqrt{\frac{1}{N}\sum_{n=1}^{N}{\left|x_{n}\right|}^{2},}$$

(6) $$P2P=\left|Max\left(X\right)-Min(X)\right|,$$

(7) $$Cf=\frac{P2P}{RMS}$$

After signal synchronization, all signals were uniformly distributed in the angular domain. To capture localized signal characteristics, statistical features were extracted using a sliding window approach. A fixed window length of ${\;\theta }_{W}$ = 15° and step size ∆θ = 1° was used to divide the full circular path into overlapping or non-overlapping angular segments. The $\theta_{0}$ denotes the reference angle (angular offset) after phase alignment across runs. For the *k*-th window, the angular interval is defined as:

(8) $$\theta_{k}\in (\theta_{0}+\left(k-1\right)∆\theta ,{\;\theta }_{0}+\left(k-1\right)∆\theta +{\;\theta }_{W})$$

Within each angular window, local statistical features of the interpolated acceleration signals-such as RMS, Mean, and SD were extracted. For the interpolated ballbar signal, only the mean value within each window was computed. This process resulted in the construction of a feature matrix X ∈ $R^{K*D}$, where each row corresponds to an angular segment and each column represents a specific statistical feature. Corresponding output values Y ∈$\;R^{K}$ were derived from the CMM-measured workpiece profile error, using either the average or the maximum error within the same angular window. This localized input–output structure facilitates spatially resolved modeling, correlation analysis, and defect predictions along the circular trajectory.

### 4.3. XGBoost Regression Model

**Figure 3:**
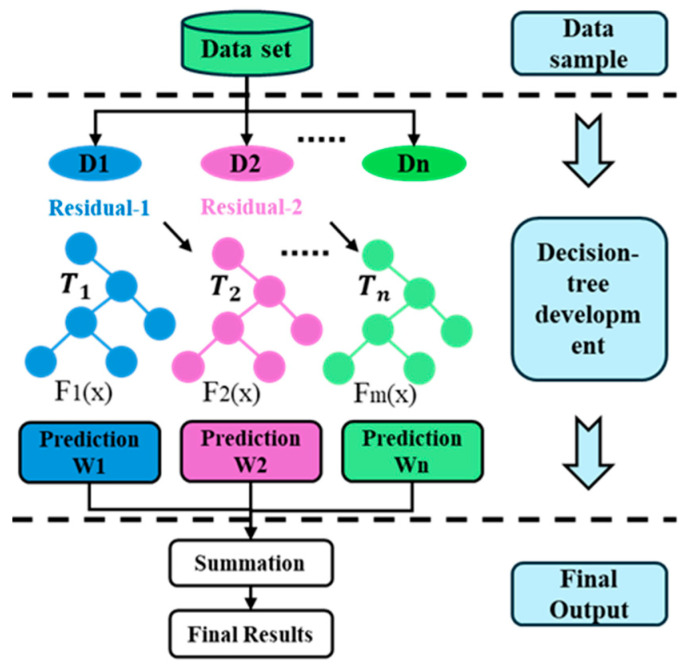
XGBoost (extreme gradient-boosting) algorithm structure. Adapted from (Maaz Amjad et al. (2022), Ref. [39]) under CC BY 4.0.

XGBoost (eXtreme Gradient Boosting) is a powerful supervised learning algorithm designed for regression and classification tasks [38]. It is built upon the principles of gradient boosting decision trees (GBDT) by integrating advanced regularization techniques, second-order optimization, and system-level enhancements for computational efficiency (Figure 3). Owing to its superior accuracy, robustness, and scalability, XGBoost has gained widespread adoption in data-driven modeling tasks, including those in manufacturing systems and condition monitoring.

In gradient boosting, models are trained sequentially, and each decision tree (learner) aims to correct the residual errors of the previous one. Formally, for a dataset $D={\left\{\left(x_{i},y_{i}\right)\right\}}_{i=1}^{n}$, where $x_{i}$ ∈ $R^{m}$ are feature vectors and $y_{i}$ ∈ R (machining error at angular position *i*) are regression targets, the ensembled prediction at step *t* is computed as:

(9) $${\hat{y}}_{i}^{\left(t\right)}=\sum_{k=1}^{t}f_{k}(x_{i}),\;f_{k}\in F$$

where $f_{k}$ denotes a regression tree from the functional space F. To learn the structure and parameters of the trees, XGBoost minimizes the following regularized loss function:

(10) $$L^{\left(t\right)}=\sum_{i=1}^{n}l\left(y_{i},{\hat{y}}_{i}^{\left(t\right)}\right)+\sum_{k=1}^{t}\Omega (f_{k}),\;f_{k}\in F$$

where l(⋅) is the loss, and the regularization term is defined by Equation (11).

(11) $$\Omega \left(f_{k}\right)=\gamma T_{k}+\frac{1}{2}\lambda \sum_{j=1}^{T_{k}}\omega_{j,k}^{2}$$

Here, l is a differentiable convex loss function (e.g., squared error) for controlling the similarities between the predictions and reference ground truths, and $\Omega \left(f\right)$ penalizes model complexity to prevent overfitting. T is the number of leaves in a tree, $w_{j}$ is the score of the *j*-th leaf node, and γ, λ are regularization parameters controlling model complexity. To optimize the computation efficiency, XGBoost uses a second-order Taylor expansion of the loss function, yielding to the simplified objective at iteration *t*:

(12) $$L^{\left(t\right)}\approx \sum_{i=1}^{n}\left[g_{i}f_{t}\left(x_{i}\right)+\frac{1}{2}h_{i}f_{t}{\left(x_{i}\right)}^{2}\right]+\Omega \left(f_{t}\right)$$

where $g_{i}=\;{\left.\frac{\partial l\left(y_{i},\hat{y}\right)}{\partial \hat{y}}\;\right|}_{\hat{y}={\hat{y_{i}}}^{\left(t-1\right)}}\;$ and $h_{i}=\;{\left.\frac{\partial^{2}l\left(y_{i},\hat{y}\right)}{\partial {\hat{y}}^{2}}\right|}_{\hat{y}={\hat{y_{i}}}^{\left(t-1\right)}}$ are the first- and second-order gradients. This formulation allows more accurate approximation and faster convergence compared to standard gradient boosting methods. To construct each tree, XGBoost selects each split which divides data into left (L) and right (R) branches by minimizing the expected gain in the loss function (Equation (13)), where the gain is calculated as:

(13) $$Gain=\frac{1}{2}\left[\frac{G_{L}^{2}}{H_{L}+\lambda }+\frac{G_{R}^{2}}{H_{R}+\lambda }+\frac{{\left(G_{L}+G_{R}\right)}^{2}}{H_{L}+H_{R}+\lambda }\right]-\gamma$$

where G and H denote the sums of gradients and Hessians in each child node. This ensures that only statistically meaningful splits are retained, and trees are automatically regularized during construction.

XGBoost distinguishes itself with a number of performance-focused improvements: parallelized tree construction and feature block caching; Sparsity-aware learning for handling missing values; weighted quantile sketch for approximate split finding on continuous data; shrinkage (learning rate control) to slow down updates and prevent overfitting, and column subsampling to reduce correlation among trees and improve generalization [38,40]. These properties make XGBoost highly efficient for large-scale structured data.

In this study, XGBoost is employed as the primary model to predict per-point machining error on circular trajectories based on features derived from both vibration signals and ballbar data. Specifically, each angular point (out of 360 per experiment) is treated as an individual sample. The feature vector for each point consists of: Time-domain vibration descriptors and ballbar-based interpolated path error at the same angular position. The target value is the CMM-measured radius error after machining. This setting constitutes a high-resolution, fine-grained regression task, where XGBoost’s capability to multi-sensor, mixed-type features with distinct physical meanings and to capture local nonlinear patterns is particularly advantageous. Additionally, the algorithm’s gain-based characteristics provides valuable interpretability, allowing us to identify which vibration or geometric characteristics most significantly affect machining accuracy.

Enhanced XGBoost variants with Bayesian hyperparameter optimization have been successfully applied to ultrasonic defect detection in concrete-filled steel tube (CFST) structures [41]. In this work, we adopt a standard XGBoost regressor with grid-based hyperparameter tuning, which is adequate given the moderate feature dimensionality and dataset size in the present robotic machining application, and keeps the model complexity compatible with shop-floor deployment.

### 4.4. Baseline Model: MLP

**Figure 4:**
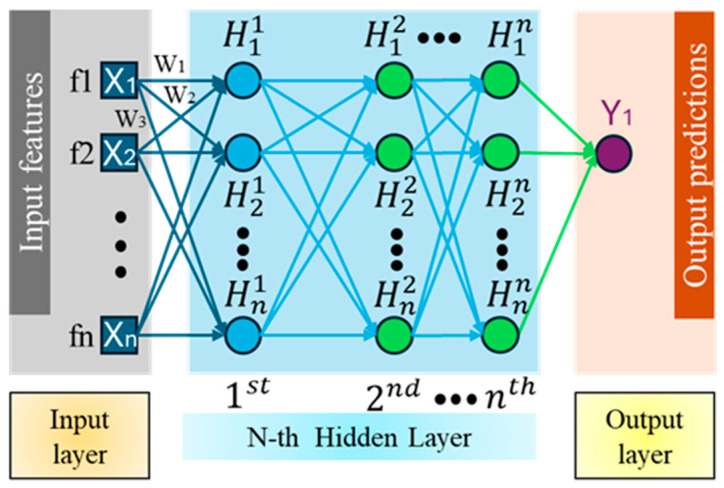
Structure of the MLP regression mechanism.

To benchmark the performance of the proposed XGBoost model, a Multilayer Perceptron (MLP) was implemented as a baseline regression model. MLPs are capable of approximating complex nonlinear mappings between input features and output targets. Unlike decision tree ensembles (e.g., XGBoost), which rely on rule-based recursive partitioning, MLPs learn smooth, continuous functions via iterative weight optimization. An MLP consists of an input layer, one or more hidden layers, and an output layer (Figure 4) [42]. Each layer is composed of multiple neurons, where each neuron performs an affine transformation followed by a nonlinear activation function. For a single hidden-layer MLP, the output prediction can be expressed as:

(14) $$\hat{y}=\phi^{\left(2\right)}(w^{\left(2\right)}\cdot \phi^{\left(1\right)}\left(w^{\left(1\right)}x+b^{\left(1\right)}\right)+b^{\left(2\right)})$$

where x∈$R^{m}$ is the input feature vector (e.g., vibration and ballbar features), $w^{\left(1\right)}$, $w^{\left(2\right)}$ and $b^{\left(1\right)}$, $b^{\left(2\right)}$ are learnable weights and biases, $\phi^{\left(1\right)}$, $\phi^{\left(2\right)}$ are nonlinear activation functions (e.g., ReLU), $\hat{y}$ ∈ R is the scalar output (predicted machining error). In this study, the MLP architecture consisted of multiple hidden layers, using the ReLU activation function. The output layer was linear to suit regression tasks. The model was trained using the Adam optimizer with mean squared error (MSE) loss function.

While MLPs are universal approximators and can in principle learn any continuous function, their practical performance is sensitive to architectural and optimization choices (e.g., number of layers and neurons, learning rate, regularization) [43]; The amount of data required for reliable generalization depends on model capacity; when capacity is large and training data are limited, overfitting can occur and degrade performance in practice Moreover, MLPs have weaker native interpretability, making feature-level insights less direct without post hoc tools [44]. Despite these drawbacks, MLPs remain a strong baseline for nonlinear regression tasks and serve as a comparative benchmark against XGBoost. Both models were trained and evaluated using the same feature inputs and data partitions to ensure a fair performance comparison, as presented in Section 5.

### 4.5. Baseline Model: Random Forest (RF)

In addition to neural network–based models such as MLP and gradient boosting methods like XGBoost, this study also considers RF as a bagging-based ensemble baseline that offers strong performance and robustness with modest tuning. Unlike boosting—where trees are added sequentially to correct residuals—RF trains many decision trees independently on bootstrap resamples of the training set and uses feature subsampling at each split to decorrelate trees and improve generalization. While MLP and XGBoost are highly expressive and can be sensitive to hyperparameter settings, especially on small datasets, these models remain widely used due to their strong regression capabilities. In contrast, RF offers a more stable and interpretable alternative with low variance and strong baseline performance.

RF constructs an ensemble of decision trees trained on bootstrapped subsets of the training data, applying feature bagging at each node to further reduce correlation among trees [45,46]. The fundamental idea is to average the predictions of multiple weak learners to obtain a robust final estimator. For regression tasks, the RF model outputs the mean prediction across all trees:

(15) $$\hat{y}=\frac{1}{T}\sum_{m=1}^{T}h_{m}(x)$$

where $h_{m}(x)$ denotes the prediction from the *m*-th tree, and T is the total number of trees. At each node, only a randomly selected subset of features is considered, promoting model diversity and improving generalization capability.

During training, each decision tree is constructed by recursively splitting the feature space to minimize the mean squared error (MSE) within child nodes. Equivalently, at any node S with $n_{S}$ samples and response mean ${\bar{y}}_{S}$, the impurity is

(16) $$MSE(S)=\frac{1}{ns}\sum_{i\in S}^{n}{\left(y_{i}-{\bar{y}}_{S}\right)}^{2}$$

and the split is chosen to maximize the reduction in this impurity (variance) over the left/right child nodes.

The strength of RF lies in its low sensitivity to hyperparameters, its resistance to noise and overfitting, and its ability to naturally assess feature importance, which is useful for model interpretation [47]. In the context of this study, RF provides a strong nonparametric baseline to validate the consistency of trends observed in MLP and XGBoost. While RF may not reach the peak performance of highly tuned deep models, its robustness and stability make it a valuable component in the comparative modeling framework.

### 4.6. Hyperparameter Optimization

All three regressors (XGBoost, MLP and RF) were implemented in Python using scikit-learn and the xgboost library. Hyperparameters were optimized separately under protocol A (Leave-one-run-out (LORO)) and protocol B (a 70:30 hold-out with five-fold cross-validation on the training split to characterize point-level performance), while always preserving a strict separation between the data used for model selection and the data used for final testing.

Under protocol A, hyperparameters are instead tuned using grouped K-fold cross-validation at the machining-run level. In this scheme, all angular samples from the same run are always assigned to the same fold, so that training and validation sets remain disjoint at the run level and no trajectory is split across folds. For all three models, we apply a randomized search over the same hyperparameters as in protocol B (Table A1) [48], but with grouped folds defined by run_id. The configuration with the lowest mean RMSE across the grouped folds is retained as the protocol A setting for each model. These settings are then used in the protocol A evaluation, where for each fold, the model is retrained from scratch on all runs except the held-out run and tested once on that held-out run.

Under protocol B, the data are first split once into a 70% training subset and a 30% hold-out test subset using a fixed random seed. Hyperparameter tuning is then performed only on the 70% training portion using RandomizedSearchCV with 5-fold cross-validation and RMSE as the scoring metric. For XGBoost, 108 random hyperparameter configurations are evaluated; for the MLP and RF baselines, 50 configurations are evaluated for each model. The candidate ranges explored for each hyperparameter, together with the final selected settings, are summarized in Table A1 in Appendix B [47,49]. RandomizedSearchCV is instantiated with a fixed random_state so that both the sampling of configurations and the shuffling of folds are reproducible. Within each cross-validation fold, early stopping is applied only on validation subsets drawn from the training folds (for example, via early_stopping_rounds in XGBoost and the built-in early-stopping mechanism in MLP), and no samples from the external 30% test subset are used for early stopping or model selection. After selection, each model is refit on the full 70% training subset using its best configuration and evaluated once on the external 30% test subset.

## 5. Training and Evaluation

**Figure 5:**
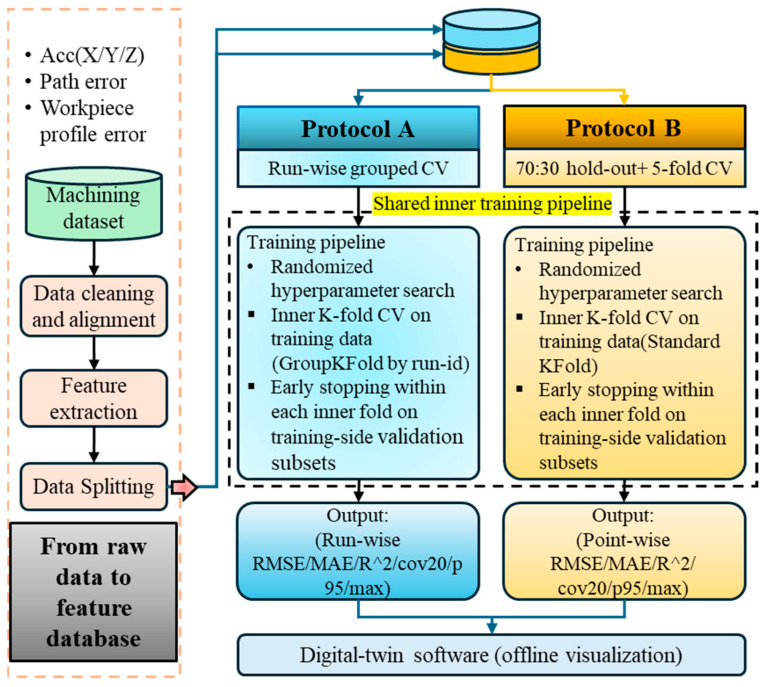
End-to-end workflow from raw machining data to XGBoost model evaluation and digital-twin visualization using two complementary protocols (run-wise grouped CV and 70:30 hold-out).

An overview of the XGBoost/MLP/RF-based trajectory error prediction framework full workflow from data capture to model inference and integration into the digital-twin frontend is illustrated in Figure 5. The process commences with machining database development, during which vibration signals acquired during machining, along with circular trajectory deviation data measured using a ballbar device, are statistically and visually analyzed to reveal underlying patterns and identify anomalies. Subsequently, data preprocessing is performed to eliminate outliers and enhance signal quality. This stage involves filtering and synchronization of the vibration signals, as well as interpolation of the ballbar-measured radial deviations to achieve alignment at 360 uniformly spaced angular positions for each experimental run. Following preprocessing, input features (e.g., RMS, SD) are constructed by extracting key descriptors from the vibration signals and combining them with the corresponding interpolated pre-machining ballbar deviations at each angular position.

For model development, two complementary protocols in parallel are used: Protocol A to quantify cross-run generalization and a 70:30 hold-out with five-fold cross-validation on the training split to characterize point-level performance (protocol B) under matched conditions. Protocol A partitions data by run, holding out one complete run per fold to eliminate cross-run correlations and quantify generalization to unseen runs; This evaluation setting is closely aligned with the anticipated deployment conditions, and thus provides more conservative, and hence more externally credible, estimates of generalization performance.

In contrast, the protocol B uses a point-level random split to provide an engineering baseline under matched operating conditions, enabling fair model comparisons on an identical partition and offering a rapid estimate of achievable performance within the training context, albeit with potentially optimistic figures due to weak within-run correlations. Using both protocols yields comparable baselines and practical reproducibility while providing a robust assessment of generalization across operating conditions. The test split in each protocol is used exclusively for final evaluation and never participates in fitting or selection to prevent leakage. Model performance is evaluated using standard regression metrics (Section 5.3). Upon completion of training, the accepted model is evaluated on the testing dataset to validate its predictive performance. The entire workflow is implemented and visualized within a LabVIEW-based digital twin system, which facilitates real-time data acquisition, model inference, and visualization of machining errors.

### 5.1. Data Splitting Strategy

Two complementary evaluation protocols are considered, as summarized in Figure 5. Protocol A applies a leave-one-run-out scheme grouped by machining run, whereas Protocol B uses a single 70:30 point-level split with a fixed random seed.

Under protocol A, samples are grouped by run_id and each outer fold trains on all but one complete machining run, which is held out for testing. All angular samples from the same physical trajectory therefore appear entirely in either the training set or the test set, mimicking deployment on a new workpiece and providing a conservative, deployment-oriented assessment of cross-run generalization. Under protocol B, all samples are pooled and a single 70:30 split is drawn at the point level, yielding an optimistic within-run scenario that serves as a baseline for attainable point-level accuracy under matched conditions. The common inner training pipeline and hyperparameter optimization procedure shared by both protocols are described in Section 4.6.

### 5.2. Model Training Protocol

All models were implemented and trained using Python 3.9, with core libraries including scikit-learn, XGBoost and NumPy. XGBoost, MLP and RF were trained under a uniform experimental protocol. Four input configurations are evaluated exactly as defined in Appendix C (Table A2): F16 (acceleration statistics plus the path error measured by the ballbar), F15 (acceleration only), F7-A and F7-B (compact acceleration features plus ballbar path error), and F6-A and F6-B (compact acceleration only). Unless otherwise stated, headline results use the F16 configuration.

For each evaluation protocol, the hyperparameter configurations selected in Section 4.6 (and summarized in Table A1) are treated as fixed. Given a particular protocol and feature configuration, the training pipeline proceeds as follows. The input features are assembled at the angle-node level, the target variable is the pointwise workpiece profile error expressed in micrometers, and any required feature scaling is fit on the training portion only and then applied to both training and test samples through the same preprocessing pipeline. Under Protocol B, each regressor is trained once on the 70% training subset using its best configuration and then evaluated once on the 30% hold-out subset. Under protocol A, grouped cross-validation is applied: for each fold, the model is retrained from scratch on all runs except the held-out run, using the Protocol-A hyperparameters from Section 4.6, and predictions are computed for all samples in the held-out run. Out-of-fold predictions from all folds are concatenated to obtain a full set of protocol A predictions, while per-run statistics are retained for subsequent analysis.

### 5.3. Evaluation Metrics

Evaluation follows the two complementary modes defined in Section 5.1. Under protocol A, samples are grouped by run_id and evaluated via grouped cross-validation by run: for each fold, an entire machining run is held out as the test set, preprocessing is fit on the remaining runs only, and predictions are computed for all samples in the held-out run. After all folds, the out-of-fold predictions on held-out runs are concatenated to compute global metrics, while per-run summaries are retained to analyze inter-run variability. Under protocol B, a single point-level split with a fixed random seed produces 70% training and 30% test; the models are trained on the 70% training subset using the hyperparameter configurations selected in Section 4.6 and then evaluated once on the external 30% test subset. Across both modes, the test portions never participate in fitting or model selection.

In this study, the performance of the XGBoost algorithm in predicting circular workpiece profile error has been evaluated using three statistical metrics: RMSE, mean absolute error (MAE), and the coefficient of determination ($R^{2}$). The RMSE is defined as the square root of the mean of the squared differences between observed and predicted values, thereby emphasizing larger errors. The MAE represents the average of the absolute differences between the predicted and observed values. The $R^{2}$ indicates the degree of correlation between predicted and actual values; a value of 1 signifies a perfect match. Let $y_{i}$ denote the actual (reference ground truth) value, ${\hat{y}}_{i}$ stands for the predicted value, and $\bar{y}$ reflects the mean of the actual values over all N data samples. These statistical metrics are computed using the following equations [50]:

(17) $$RMSE=\sqrt{\frac{1}{N}\sum_{i=1}^{N}{\left(y_{i}-{\hat{y}}_{i}\right)}^{2}}$$

(18) $$MAE=\frac{1}{N}\sum_{i=1}^{N}\left|y_{i}-{\hat{y}}_{i}\right|$$

(19) $$R^{2}=1-\frac{\sum_{i=1}^{N}{\left(y_{i}-{\hat{y}}_{i}\right)}^{2}}{\sum_{i=1}^{N}{\left(y_{i}-{\bar{y}}_{i}\right)}^{2}}$$

To align with shop-floor tolerances, three additional error metrics are reported: (1) coverage within ±20 µm, defined as the fraction of points with $\left|e_{i}\right|\leq 20\;µm,\;$ where $e_{i}={\hat{y}}_{i}-y_{i}$; (2) the 95th percentile of $\left|e_{i}\right|$(p95) to summarize the tail; (3) the maximum absolute error $max\left|e_{i}\right|$. To further analyze spatial structure and local heteroscedasticity, the circular trajectory is partitioned into equal angular segments. Specifically, the range [0°, 360°] is divided into 30° bins, and angle-wise behavior is assessed within each segment. For every bin, cov20 is reported along with its corresponding Wilson score interval at z = 1.96 to quantify statistical uncertainty.

## 6. Results and Comparative Analysis

### 6.1. Model Performance—Protocol A

**Figure 6:**
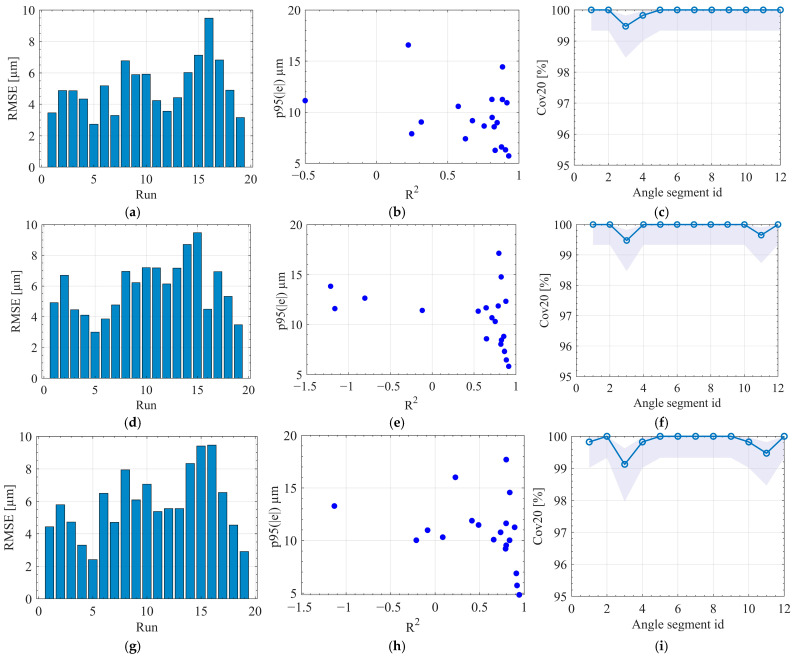
Prediction performance on workpiece profile error for XGBoost, MLP, and RF. Panels (**a**–**c**) show XGBoost results: (**a**) per-run RMSE under protocol A, (**b**) relationship between p95($\left|e\right|$) and R^2^, (**c**) Cov20 distribution across angle segments. Panels (**d**–**f**) show MLP results: (**d**) per-run RMSE under protocol A, (**e**) relationship between p95($\left|e\right|$) and R^2^, (**f**) Cov20 distribution across angle segments. Panels (**g**–**i**) show RF results: (**g**) per-run RMSE under protocol A, (**h**) relationship between p95(|e|) and R^2^, (**i**) Cov20 distribution across angle segments. Here e denotes prediction error, p95($\left|e\right|$) is the 95th percentile of absolute error, Cov20 is the percentage of points with |e| ≤ 20 µm, and protocol A is leave-one-run-out.

Using the full set of 16 features (Table A2, Appendix C), the predictive performance of XGBoost, MLP, and RF was comprehensively evaluated. All results in Figure 6 are obtained with protocol A, where each fold trains on all but one run and is evaluated only on the held-out run; all metrics are computed on these unseen runs. Under this setting, XGBoost delivers the strongest and most stable generalization (Figure 6a–c). Its per-run RMSEs concentrate near the lower end of the observed range, and the scatter in the p95($\left|e\right|$)–R^2^ plane clusters in the quadrant of small p95($\left|e\right|$) with high R^2^, indicating that both average error and tail risk are well controlled. Cov20, defined as the percentage of points with |e| ≤ 20 µm, remains close to 99–100 percent along angle segments, with only shallow dips and narrow Wilson intervals. RF is competitive but less consistent (Figure 6d–f). Several runs shift to mid-range RMSE, the p95($\left|e\right|$) distribution shows a broader tail, and Cov20 is slightly less flat across segments. The MLP performs weakest (Figure 6g–i). Multiple runs exhibit low or negative R^2^, RMSE dispersion is widest, and p95($\left|e\right|$) is visibly larger for several cases, which suggests sensitivity to run-level shifts and limited robustness without additional regularization or feature engineering. Across models, angle-segment sensitivity is small, supporting the effectiveness of the synchronization, direction and phase alignment, and angle-domain resampling pipeline. To complement the quantitative comparisons, Appendix D presents a representative example of the predicted and measured workpiece profile error for a typical machining run.

**Figure 7:**
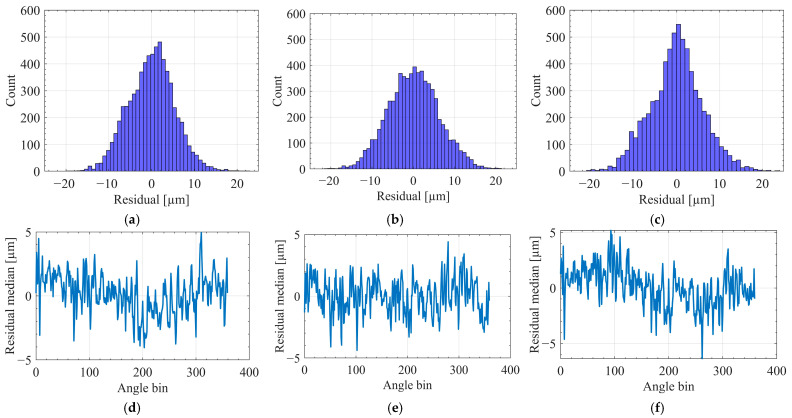
Residual distribution and median residual values across all runs using the protocol A. Subfigures (**a**–**c**) display residual histograms for XGBoost (**a**), MLP (**b**), and RF (**c**), respectively. Subfigures (**d**–**f**) show the median residual as a function of angle bin for each corresponding method.

Figure 7 presents the residual structure under the protocol A across 19 runs. Figure 7a–c display histograms of the residuals, demonstrating that all three models yield distributions cantered near zero with approximately symmetric shapes. This pattern indicates the absence of global bias following synchronization, phase alignment, and angle-domain resampling. Among the models, XGBoost produces the narrowest distribution with the lightest tails, followed by MLP, while RF exhibits the widest spread and the heaviest tails. This trend aligns with the per-run performance metrics discussed previously, where XGBoost achieved the lowest RMSE and smallest p95($\left|e\right|$).

Figure 7d–f show the median residuals plotted against angle bins, aggregated over held-out folds. The fluctuations around zero suggest that dominant systematic errors have been effectively removed, and that the remaining deviations are predominantly local. XGBoost again demonstrates the highest stability, characterized by low-amplitude, high-frequency variations with minimal sustained drift. MLP exhibits slightly larger fluctuations, whereas RF presents occasional low-frequency drifts and spikes, reflecting greater sensitivity to run-specific dynamics. In conjunction with the high Cov20 reported earlier, these angle-resolved profiles confirm that the majority of points remain within the ±20 µm quality band, with deviations concentrated in a limited number of angle segments.

**Figure 8:**
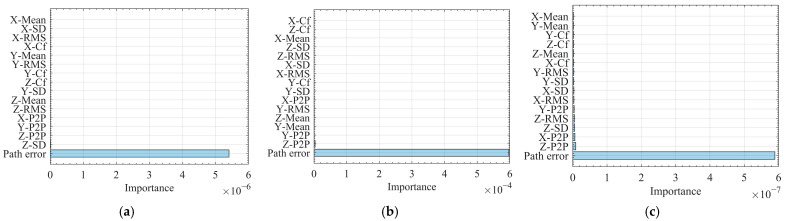
Feature importance distribution calculated by XGBoost (**a**), MLP (**b**), and RF (**c**) across all runs using protocol A. Feature-importance scores are shown on a normalized linear scale (no log transformation).

To further evaluate the behavior of the XGBoost model, Figure 8 summarizes the feature importance under protocol A for XGBoost (a), MLP (b), and RF (c). Across all three models, the ranking is notably consistent: the path error feature dominates by one to two orders of magnitude, whereas vibration-based statistics (RMS, SD, crest factor) and axis-wise peak-to-peak or mean terms contribute only marginally. This pattern indicates that the ballbar-derived path deviation captures most of the variance required to predict the workpiece profile error, while inertial cues act primarily as minor refinements—enhancing local segments affected by dynamic disturbances but not governing overall accuracy.

Small differences emerge within the vibration-based features: dispersion metrics along the Z-axis (e.g., Z-SD) tend to receive slightly higher weights than their X/Y counterparts, consistent with the earlier angle-wise residual dips that suggest localized out-of-plane dynamics. Although absolute importance values vary among algorithms due to differing internal scales, the stable feature ordering provides the most reliable insight.

In this study, feature importance scores are computed using the gain metric in XGBoost, which measures the average reduction in training loss achieved by splits on a given feature. Because split-based gain can overestimate the importance of features with many effective split points (for example, continuous variables with fine resolution) [51], the rankings in Figure 8 are interpreted only qualitatively and are not used in isolation for strong claims. Even under a conservative interpretation, the ballbar-derived path error feature was found to be overwhelmingly dominant; its gain exceeded that of any individual vibration statistic by more than an order of magnitude. This result suggests that the learned model is primarily geometry-anchored, with the circular deviation measured by the ballbar serving as the principal source of predictive power. In contrast, the Y- and Z-axis vibration statistics function as secondary dynamic indicators, contributing mainly to the refinement of the error distribution tails and coverage metrics. The conclusion that the ballbar path error constitutes the dominant predictive feature, with only a marginal contribution from selected vibration statistics, is further substantiated by the feature ablation analysis in Section 6.3 and the profile-level comparisons presented in Appendix D.

In addition to the graphical results presented in Figure 6 and Figure 7, the numerical comparison in Table 2 provides a quantitative assessment of model performance under protocol A. When individual runs are held out for testing, XGBoost-A achieves an RMSE of 5.4 µm and an MAE of 4.3 µm, with $R^{2}=0.806$, Cov20 = 99.94%, p95=10.4 µm, and a maximum absolute error of 23 µm. In comparison, MLP-A and RF-A yield RMSE values near 6.1 µm, $R^{2}\approx 0.75$, and $p_{95}$ values in the range of 11.9–12.2 µm. This corresponds to an RMSE reduction of approximately 11% and a 1.5–2.0 µm improvement in $p_{95}$ for XGBoost relative to the strongest baseline, while all three models maintain similarly high coverage within the ±20 µm quality band. Given that all metrics are reported at micrometer resolution, these differences are practically significant. The results confirm that XGBoost offers the most favorable balance between average accuracy and tail error suppression when generalization to unseen runs is required.

### 6.2. Model Performance—Protocol B

**Figure 9:**
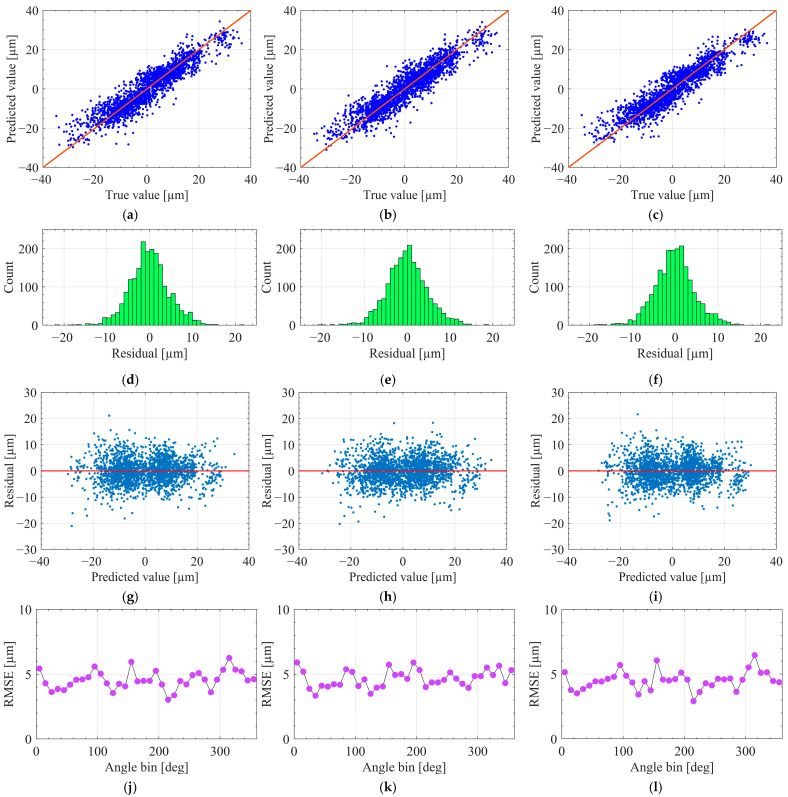
Prediction performance of workpiece profile error under the protocol B for XGBoost (**a**,**d**,**g**,**j**), MLP (**b**,**e**,**h**,**k**), and RF (**c**,**f**,**i**,**l**). Subfigures (**a**–**c**) show the correlation between predicted and true values, with a y = x reference line. Panels (**d**–**f**) present residual histograms, highlighting the symmetry and dispersion of prediction errors. Subfigures (**g**–**i**) plot residuals against predicted values, revealing no apparent heteroscedasticity or systematic bias. Panels (**j**–**l**) display the RMSE of test data distributed across angular bins along the circular path. Together, these visualizations provide a comprehensive assessment of each model’s accuracy, residual behavior, and angular consistency under independent evaluation.

Under protocol B, the dataset was randomly partitioned at the trajectory-point level, with 70% of the samples used for training and the remaining 30% reserved for testing, without stratification in the angular domain. Figure 9 presents the prediction results obtained under this setting.

The top row compares predicted and measured path errors. All three regressors demonstrate a clear linear correlation with the ground truth. Among them, XGBoost produces the tightest cluster along the y = x reference line, yielding the highest $R^{2}$ and an RMSE of approximately 5 µm on the test set. MLP yields a slightly wider spread of points, whereas RF exhibits the largest dispersion and more pronounced deviations from the diagonal, indicating reduced nonlinear fitting capacity when trained on the same features and sample budget.

The second row presents residual histograms for each model. All distributions are approximately symmetric and bell-shaped, centered near zero, indicating that the random 7:3 train-test split does not introduce systematic bias into the predictions. The third row shows residuals plotted against predicted values. No funnel-shaped patterns or pronounced nonlinear trends are observed, and the residual variance remains relatively stable across most of the prediction range, with a slight tendency toward underestimation at the largest-magnitude errors. The bottom row reports RMSE values across angular segments along the circular trajectory. Although local fluctuations are present, none of the models exhibits consistent degradation within specific angular sectors. This behavior is consistent with the high Cov20 values and is further reflected in the reported 95th percentile error (p95) and maximum absolute error (Max), which together characterize the tails of the error distribution.

The protocol B statistics in Table 2 further reinforce the preceding observations by providing a concise numerical summary of point-level prediction performance under matched operating conditions. All three models achieve comparable accuracy: XGBoost-B and RF-B both attain an RMSE of 4.6 µm, while MLP-B reports 4.7 µm. The corresponding $R^{2}$ values are 0.8579, 0.8599, and 0.8514, respectively. Coverage within the ± 20 µm tolerance band is consistently high, ranging from 99.9% to 100%, and the p95 values lie within a narrow interval of 9.3–9.5 µm. Maximum absolute errors vary slightly, from 20.2 µm to 21.5 µm. These results indicate that, when training and test samples originate from the same runs, XGBoost, MLP, and RF all interpolate the trajectory with nearly indistinguishable global accuracy. Differences among the models are limited to minor variations in tail behavior and peak error, underscoring their similar performance under intra-run conditions.

In contrast, protocol A discussed previously provides a more stringent test of generalization, as it evaluates model performance across independent runs that may differ in dynamic characteristics or operational conditions. Under this setting, XGBoost demonstrates substantially better performance than the other models, achieving lower RMSE, higher $R^{2}$, and smaller p95 values. These discrepancies highlight that the performance gap under protocol A arises primarily from run-to-run variability rather than intrinsic model limitations. Consequently, protocol B serves as an optimistic within-run reference case, illustrating the best-case accuracy when the test distribution closely matches the training data, whereas Protocol A reveals how each model responds to distribution shifts across independent machining runs under realistic deployment scenarios.

Taken together, these findings support the selection of XGBoost as the preferred engine for digital twin modeling. It offers competitive accuracy under ideal, matched conditions and exhibits markedly stronger robustness when deployed across previously unseen runs—making it the most suitable choice for practical, shop-floor implementations where generalization is critical.

### 6.3. Feature Importance Analysis

While Section 6.1 and Section 6.2 have established that the ballbar-derived radius error is the dominant predictive feature and that acceleration-based statistics contribute only modest additional value, they do not identify which specific vibration features are informative or how many are actually necessary. Given that the digital twin is intended for deployment on shop-floor hardware, considerations such as signal processing time, feature extraction latency, and model complexity must be balanced against prediction accuracy.

To address this, a feature importance and ablation study is conducted using XGBoost with four nested feature sets comprising 16, 15, 7, and 6 variables, as defined in Table A2 (Appendix C). These configurations are constructed to progressively remove the ballbar path error and lower-order vibration descriptors, spanning from a full hybrid set (F16) to minimal vibration-only sets (F7, F6) in order to balance predictive power against sensor and preprocessing complexity. Feature importance is computed from the XGBoost model. By comparing prediction performance and learned feature importances across these configurations, the analysis identifies which variables are essential, how much the feature set can be compressed without compromising accuracy, and the degree to which vibration features provide complementary information beyond the ballbar signal.

**Figure 10:**
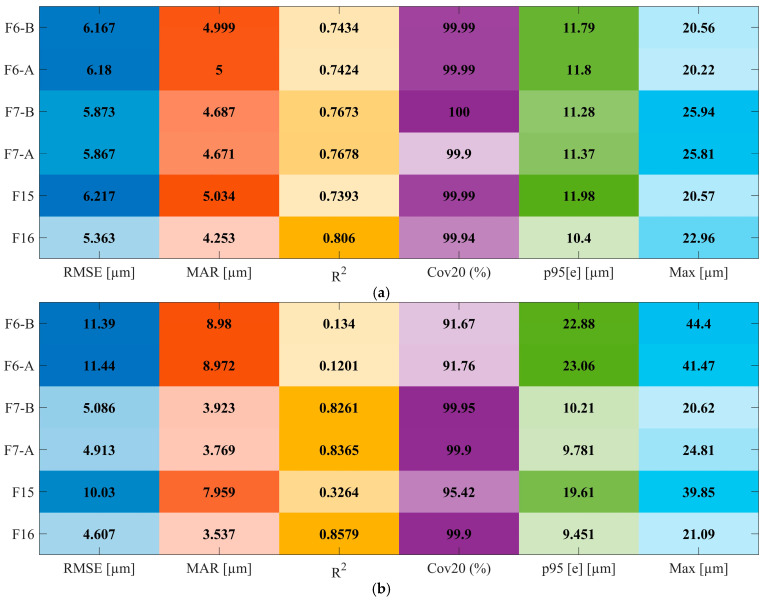
Effect of Feature Set Selection on XGBoost Performance for Pointwise Radius-Error Prediction. Each panel presents a comparison of six feature configurations (F16, F15, F7-A, F7-B, F6-A, F6-B): (**a**) results obtained under Protocol A; (**b**) results obtained under Protocol B. Cells report RMSE, MAE, R^2^, Cov20, p95 and Max. Cell colors indicate the relative magnitude of each metric within a column, with darker shades corresponding to larger values.

Figure 10 summarizes the XGBoost performance obtained using the six feature configurations under protocols A and B. Under Protocol B, which reflects favorable intra-run conditions, the full feature set (F16) achieves the best performance, with a RMSE of approximately 4.6 µm and a R^2^ of approximately 0.86. Both compact configurations, F7-A and F7-B, yield comparable results, with RMSE values in the range of 4.9–5.1 µm and R^2^ between 0.83 and 0.84. In contrast, removal of the ballbar path error (F15) results in a substantial degradation in performance, with RMSE nearly doubling to approximately 10 µm, R^2^ decreasing to 0.33, and both the 95th percentile error (p95) and maximum absolute error nearly doubling.

The vibration-only configurations, F6-A and F6-B, exhibit even poorer performance, with R^2^ values around 0.12–0.13, although cov20 remains above 90%. These results confirm that the ballbar-derived radius error is essential for accurate pointwise prediction, while additional acceleration descriptors contribute only marginal improvements once the path error is included.

Under the more demanding protocol A, shown in Figure 10a, which assesses cross-run generalization, the relative ranking of feature sets remains consistent, though performance differences are less pronounced. The F16 configuration again yields the best results (RMSE ≈ 5.4 µm, R^2^ ≈ 0.81), with F7-A and F7-B remaining within approximately 0.5 µm in RMSE and within 0.04 in R^2^, while maintaining nearly identical cov20 and p95 levels. Although the F15 and F6 configurations continue to show elevated RMSE and reduced R^2^, the performance deterioration is less severe than under Protocol B. This suggests that, under cross-run conditions, the benefits of enriched vibration statistics are partially obscured by inter-run variability. Furthermore, the similarity between F7-A and F7-B indicates that low-order descriptors (mean and standard deviation) and high-order descriptors (peak-to-peak and crest factor) capture largely redundant aspects of vibration behavior when used alongside the ballbar signal.

**Figure A3:**
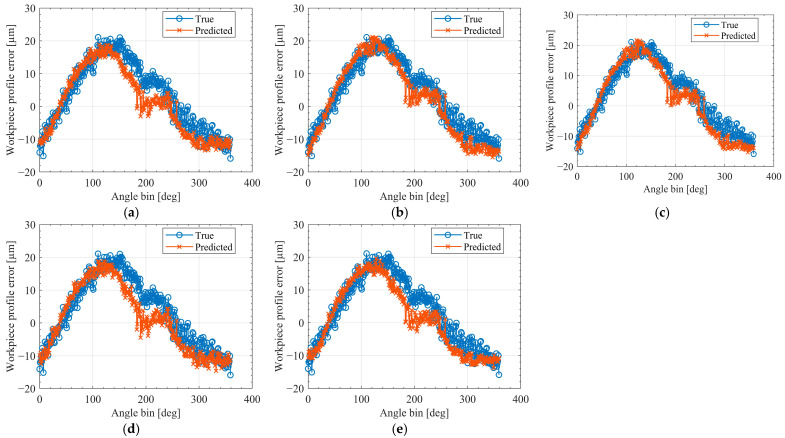
Predicted and measured workpiece profile error for one representative run using XGBoost with input feature sets F15 (**a**), F7-A (**b**), F7-B (**c**), F6-A (**d**), and F6-B (**e**).

To complement the aggregate metrics presented in Figure 10, Appendix D (Figure A3) provides a pointwise comparison between the predicted and measured workpiece profile errors for a representative run under the F15, F7-A, F7-B, F6-A, and F6-B feature configurations. The hybrid sets incorporating ballbar input (F7-A and F7-B) closely track the measured curve, exhibiting only minor local deviations. In contrast, the vibration-only configurations (F6-A and F6-B) display markedly larger discrepancies, particularly near the peak region and along the descending flank. This qualitative behavior aligns with the quantitative trends observed in the ablation study and underscores the importance of the ballbar-derived path error as a critical geometric reference for accurate trajectory-level prediction.

In summary, the ablation results demonstrate that most of the predictive capability can be preserved using a compact feature set that includes the ballbar path error and six selected vibration descriptors. The F7-A and F7-B configurations reduce the number of input variables from 16 to 7, thereby lowering the signal-processing and feature-extraction demands by more than half, while incurring only a modest loss in RMSE and R^2^ across both protocols. In contrast, exclusion of the ballbar feature and reliance solely on vibration statistics leads to a pronounced reduction in explanatory power. These findings support the use of the ballbar-derived radius error as the primary predictor in digital twin models, with supplementary vibration features incorporated when computational resources allow.

### 6.4. Interpretation of Error Sources and Deployment Implications

The findings of Section 6.1, Section 6.2, Section 6.3 indicate a structured division of roles between the two sensing modalities. The ballbar-derived circular path deviation functions as a quasi-static geometric anchor, accounting for the majority of the variance in the workpiece error. In contrast, the acceleration-based descriptors capture dynamic effects, such as tool–workpiece interaction and local structural compliance. Within this framework, the XGBoost model may be interpreted as a geometry-plus-dynamics surrogate, wherein the ballbar feature defines the baseline error field, and the vibration descriptors provide localized corrections that refine the residual distribution and mitigate extreme deviations at specific angular positions.

This interpretation also clarifies the divergence observed between regression metrics and coverage indicators in the feature ablation study. Upon removal of the ballbar information, regression performance degrades substantially, underscoring the necessity of geometric observability for pointwise prediction. Nevertheless, coverage within the ±20 µm tolerance band often remains relatively high. This indicates that vibration-only configurations primarily function as conservative anomaly detectors, which are inclined to signal large deviations. In contrast, hybrid configurations—retaining both the ballbar feature and a compact subset of vibration descriptors—enable the generation of high-fidelity error maps and afford tighter control of the 95th percentile error (p95) and maximum absolute error across runs.

From a quality control standpoint, these findings support a two-tiered deployment strategy. For tasks aimed at determining whether a given machining run meets a specified tolerance envelope, simplified sensing schemes employing reduced feature sets already yield robust binary pass/fail decisions. For applications requiring dense error field predictions—such as trajectory compensation or process optimization—the hybrid regime, combining ballbar data with selected vibration features, is indispensable. The feature ablation results further suggest a practical degradation pathway: beginning with the full feature set, the system may revert to compact configurations such as F7 with only modest performance loss. In contrast, complete removal of the ballbar signal induces a qualitative transition from geometrically anchored regression to vibration-driven anomaly detection.

## 7. Digital Twin Implementation

**Figure 11:**
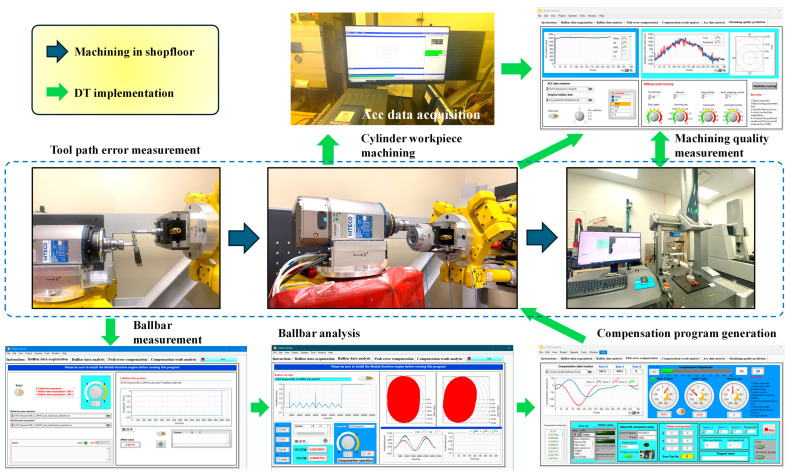
Offline digital twin implementation for robotic machining. The workflow couples shop-floor execution and sensing (**top** path) with offline digital twin computation, error prediction, compensation export, and validation (**bottom** panels), forming a closed loop that is deployment-ready for future online operation.

Section 3 presented the overall architecture and the static data pipeline. This section turns to practical operation and validation, explaining how the framework performs acquisition, analysis, prediction, compensation export, and re-inspection in an offline closed loop. The end-to-end workflow is illustrated in Figure 11. The robot executes a programmed circular path on a spindle-mounted workpiece. The selected ballbar continuously records in-plane radius variation, while three accelerometers records vibration. Acquisition is managed by a LabVIEW panel that performs channel health checks, saturation alarms, drop detection, and file writing (Acc data is currently acquired by Siemens LMS software). Each run stores a unified metadata header including date, operator, workpiece ID, nominal radius, feed rate, spindle speed, tool and fixture state, sampling rates, and ranges for all channels. All sensor streams are time-stamped at the source and saved together with a human-readable metadata file to ensure traceability and reproducibility.

After acquisition, all data are imported into the digital twin workspace. Signals are first converted to the angular domain and aligned on a common angular grid. A one-degree resolution is used by default and can be adjusted when needed. A fixed window and stride are then used to generate the feature set from the ballbar trace and the three vibration axes, including statistical measures, band-limited energy, and harmonic indicators. Interpolation methods are kept consistent across signals to avoid shape distortion. Features are standardized with the same parameters used in training in order to prevent distribution shift. The offline batch computation time per circular run is defined as the sum of synchronization, feature generation, model inference, and export. In the current implementation on a standard desktop computer, the full pipeline for a single circular run executes well below one second, and processing all 19 runs requires only a few seconds in total, which is negligible compared with the machining time per run and is sufficient for the intended offline batch-compensation workflow.

The interface provides linked visualizations: an angular plot comparing prediction and measurement, a polar error map for local peaks, and vibration spectra for all three axes. Each run reports a set of quality indicators for both model assessment and process diagnosis, including mean radius deviation, peak-to-peak error, a roundness proxy, and the distribution of per-angle residuals. The interface supports zooming on specific arc segments and annotates correspondences between dominant spectral components and path-error harmonics to help localize periodic sources.

The predicted radial deviation is converted into a radius correction table and lightly smoothed to suppress isolated spikes while preserving the dominant periodic content. The export module generates controller-ready files. For the robot cell, a FANUC LS program is produced, including a header that records the model and feature versions, a configuration digest, and paired angular correction entries. The program is designed to apply radial offsets relative to the nominal toolpath. All exported files are time-stamped and encoded with configuration checksums, enabling one-to-one traceability between machining outcomes and the corresponding digital twin state. In the present study, these compensated programs are generated and analyzed offline but are not executed on the robot during the reported experiments. As such, the numerical results focus on prediction accuracy and coverage metrics, rather than on demonstrated part improvements. Offline circular path error measurement and compensation using ballbar-derived correction tables has been implemented and experimentally validated on the same hexapod-based machining cell in prior work [35], confirming the practical feasibility of the controller interface. The XGBoost-based compensation profiles developed in the present digital twin framework are intended for deployment through this interface in future closed-loop experiments, where the resulting reduction in profile error and its run-to-run repeatability across multiple parts will be quantitatively assessed.

Robustness is enforced at several layers. The acquisition side performs range and saturation checks. The offline stage performs window completeness checks and small-gap imputation. If the accelerometer stream does not meet quality thresholds, the system degrades to a ballbar-dominant feature set and clearly marks the run in the log. All events and key parameters are recorded so that the figures and conclusions can be reproduced under the same configuration. All critical parameters are maintained in a readable configuration file, including angular resolution, window width and stride, sampling rates and interpolation settings, the ordered feature list, standardization parameters, and the model identifier with a version hash. The export module writes a configuration summary into the controller file header. A separate manifest links each machining run, digital-twin configuration, exported program, and CMM report, forming an auditable chain across batches and hardware.

Although the current workflow is offline, interfaces and message formats mirror those required for an online twin. A replay utility packages recorded data into angle blocks and emits them at a chosen rate to evaluate end-to-end timing and potential lead-angle policies in a workstation-only setting. This shadow evaluation does not require controller access. The same compensation generator is used in both modes, enabling a smooth transition to shadow and feed-forward operation once network access and safety interlocks are available. The implementation does not interact with the controller during cutting and therefore does not address fast disturbances that demand in-cycle actuation. The focus is on program-level, predict-then-compensate corrections, which are well suited to quasi-static path errors and repeatable periodic content. CMM validation confirms measurable geometric improvement under these conditions, while preserving a clear pathway toward an online twin in future work.

## 8. Discussion

The present study demonstrates that a data-driven digital twin—integrating ballbar measurements and angle-domain vibration features—can achieve micrometer-level accuracy in pointwise trajectory error prediction for robotic machining. A key finding is the pronounced contrast observed between the two evaluation protocols. Under the more realistic protocol A setting, XGBoost consistently achieves RMSE, MAE, cov20, p95 and maximum absolute error values compared to the MLP and RF baselines across all 19 runs. In contrast, when the evaluation is relaxed to a random intra-run pointwise split, these differences largely disappear, and all models exhibit similar performance. This outcome suggests that XGBoost is particularly well-suited for addressing moderate distributional shifts across machining trials, and further underscores that evaluation protocols based solely on random splits may substantially overestimate generalization capability.

The ablation and feature importance analyses clarify the complementary roles of geometric and inertial sensing within this framework. The ballbar-derived circular path deviation serves as a quasi-static geometric anchor, capturing the dominant contribution to the workpiece profile error. Once this anchor is established, vibration-based descriptors contribute moderate improvements to global regression metrics but consistently reduce the 95th percentile and maximum absolute errors, particularly in angular sectors prone to dynamic amplification and chatter-like phenomena. In this sense, the acceleration features act as localized dynamic refinements over a geometry-driven baseline, rather than functioning as standalone predictors. This interpretation aligns with prior machining studies, which report that vibration signals are highly sensitive to transient anomalies but are only indirectly related to absolute dimensional deviations.

From a quality control perspective, these findings support a two-tiered usage strategy and suggest a practical degradation path for sensing and feature allocation. For tasks requiring binary pass/fail decisions with respect to a fixed tolerance envelope, simplified feature sets—even those dominated by vibration descriptors—can serve as effective conservative anomaly detectors, as large deviations are rarely missed despite potential imperfections in regression accuracy. For applications demanding dense and precise error maps to enable trajectory compensation or feedrate optimization, the hybrid regime—combining the ballbar-derived path error with a compact subset of vibration features—is essential. Starting from the full 16-feature configuration, the system can revert to more compact variants (e.g., F7-A or B) with only minor degradation in performance. However, complete omission of the ballbar signal induces a qualitative shift, from geometrically anchored regression to vibration-driven anomaly scoring. This behavior is particularly relevant for deployment on the shop floor, where sensor availability and computational resources are often limited.

Several limitations and broader implications warrant consideration. The present experiments focus on a single family of circular interpolations within a restricted posture region, using one material, tool, and parameter set. Consequently, the learned mapping implicitly absorbs posture-dependent stiffness and process conditions rather than modeling them explicitly. While the dataset size (19 runs) is sufficient for tree-based ensemble methods, it remains limited for deep learning approaches and may constrain the model’s generalization. This challenge of data scarcity is common in ML-based manufacturing applications due to the high cost of data acquisition. Future work could address this limitation by incorporating virtual sample generation (VSG) techniques, which have been shown to significantly improve prediction performance in applications like CFRP drilling when training data is scarce [52]. Building on these ideas, future work will combine the proposed feature set and digital-twin structure with VSG or related small-sample augmentation strategies to systematically expand the effective dataset and re-evaluate generalization across trajectories, robot postures, and cutting parameters. Additionally, the uncertainty analysis primarily addresses Type A repeatability of the ballbar measurements, with only preliminary treatment of other sources of variability. The digital twin, as currently implemented, operates in an offline or quasi–real-time mode, which is suitable for batch-level compensation but not yet compatible with fully in-process correction. In its current form, the digital twin is validated through prediction metrics rather than through a complete closed-loop machining and inspection campaign. As a result, the industrial benefit is demonstrated indirectly. This is achieved through accurate trajectory-level error prediction and the capability to generate controller-compatible compensation tables, rather than by an explicit before–after comparison of compensated parts within the scope of this study. Nevertheless, offline or quasi–real-time digital twins of this nature are already practically valuable. They support capability verification for new setups, facilitate the localization of critical angular sectors for process tuning, and generate compensation files that can be deployed when production schedules permit additional machining and inspection trials.

On the same dual-hexapod platform, prior work has demonstrated that ballbar-based radius correction can effectively reduce circularity and radius deviation when implemented at the controller level, with improvements verified through CMM inspection [35]. The current implementation reuses this compensation interface, with the predicted error profile and corresponding correction tables supplied by the XGBoost model. Therefore, extending the present study to include a dedicated closed-loop experiment, in which the digital-twin-generated compensation is applied and the resulting parts are evaluated using CMM, represents a clearly defined and high-priority direction for future work, rather than a limitation to industrial applicability.

Moreover, expanding the dataset to encompass a wider range of trajectories, materials, and process conditions; enriching the sensing configuration where economically justified; and extending the modeling approach toward probabilistic regression with calibrated prediction intervals would significantly enhance the robustness of the framework. Coupled with deeper integration into the robot controller, these developments could progressively transform the system from an offline diagnostic twin into a reliable decision-support and compensation tool for high-precision robotic machining.

## 9. Conclusions and Future Work

This study presented a data-driven digital twin for pointwise prediction of circular trajectory errors in a hexapod-based robotic machining system. The approach combines synchronized ballbar measurements with angle-domain vibration features, processed through a unified signal chain for denoising, resampling, and phase alignment. An XGBoost regressor was trained to map the hybrid feature set to measured profile errors. Model performance was evaluated under a deployment-oriented leave-one-run-out protocol and a within-run random split, using multilayer perceptron and random forest as baselines.

1. Under the leave-one-run-out setting, the XGBoost model achieved micrometer-level accuracy with low RMSE and high coverage within ±20 µm, while maintaining tighter error tails than the baseline models. When training and test points were randomly split within runs, the performance of all models converged, indicating that the observed differences are primarily due to run-level distribution shifts.
2. The ballbar-derived circular deviation functions as a geometric anchor, explaining most of the error variance. Vibration features contribute local refinements, improving high-percentile and peak errors in dynamically sensitive regions. However, in the absence of geometric input, vibration-only configurations fail to reconstruct detailed error distributions and behave more as anomaly detectors.
3. Compact hybrid configurations, which retain the ballbar signal and a small number of vibration descriptors, preserve most of the predictive accuracy of the full feature set. This enables scalable deployment: simplified configurations are suitable for trajectory error compensation, while more aggressive reduction can support tolerance verification and anomaly screening under resource constraints.
4. The proposed method achieves trajectory-level accuracy using low-cost sensors, while remaining compatible with compact robotic cells. Although tested on a dual-robot hexapod for mechanical flexibility, the method can be applied to single-robot or hybrid machining setups. The trained model can be integrated into offline pipelines that generate compensation tables, enabling batch-level error correction without requiring real-time control.

Several limitations remain. The current study focuses on a specific circular trajectory, posture region, and fixed machining parameters. Generalization to broader paths, materials, and operating conditions will require dataset expansion and inclusion of relevant process descriptors. Workspace-level behavior and static compliance are only implicitly captured; incorporating stiffness models and extending the geometric anchor to three dimensions would improve robustness across varying configurations. The present validation is restricted to a single, relatively light circular milling regime (100 µm radial depth of cut). As such, the industrial generality of the proposed digital twin cannot yet be claimed beyond similar finishing-like conditions. Uncertainty analysis has so far addressed repeatability of the ballbar signal, with only a preliminary budget for other sources. A more complete uncertainty framework is needed, including contributions from calibration, fixturing, environment, and reference devices, and systematic propagation through the model. Enhancing the digital twin with predictive intervals and explicit coverage control would further improve reliability for deployment.

Finally, the current implementation operates in an offline mode. Future work will address latency, control constraints, and communication architecture to enable online prediction during machining at heavier cuts and broader operating regimes. This advancement would support the transition from shadow mode to full controller-integrated operation in high-precision robotic manufacturing. In addition, future work will construct a complete Guide to the Expression of Uncertainty in Measurement (GUM) consistent uncertainty budget once full calibration data become available, rather than limiting the analysis to the ballbar Type A component. It will also investigate how explicit stiffness models and volumetric stiffness maps can be integrated into the digital twin so that geometric and compliance related effects are treated in a unified way.

## Figures and Tables

**Table 1:** Specification of workpiece and cutter.

Category	Parameter	Value
Workpiece Material	Material Type	6061-T651 Aluminum
	Ultimate Tensile Strength (MPa)	320
	Modulus of Elasticity (GPa)	69
	Elongation at Break (%)	11
	Brinell Hardness (HB)	90–105
	Main Alloying Elements	Al–Mg–Si–Cu (AIMg1SiCu)
Cutting Tool	Tool Model	NIAGARA A345-0.500-D3-S.0-Z3
	Cutter Diameter (mm)	12.7
	Number of Cutting Edges	3
	Helix Angle (°)	45
	Overall Length (mm)	76.2
	Coating	None

**Table 2:** Summary of regression metrics for XGBoost, MLP and RF under protocol A and protocol B.

Method	RMSE [µm]	MAE [µm]	R2	Cov20	P95% [µm]	Max [µm]
XGBoost-A	5.4	4.3	0.806	99.94%	10.4	23
XGBoost-B	4.6	3.5	0.8579	99.9%	9.5	21.1
MLP-A	6.1	4.9	0.7496	99.93%	11.9	21.4
MLP-B	4.7	3.6	0.8514	100%	9.4	20.2
RF-A	6.1	4.8	0.7457	99.8%	12.2	25.9
RF-B	4.6	3.5	0.8599	99.9%	9.3	21.5

**Figure A1:**
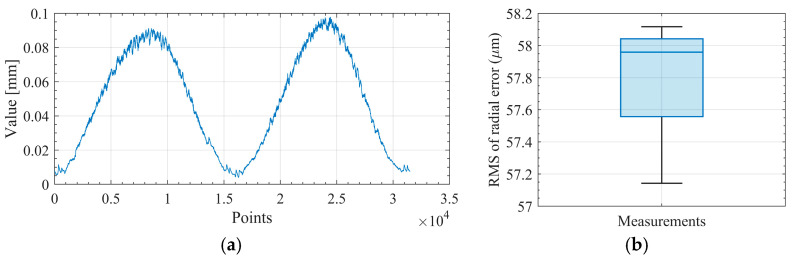
(**a**) Example angle-domain radial error from the off-line ballbar in the machining direction (x-axis denotes sample index). (**b**) Box plot of per-circle RMS over *n* = 5 repeated CCW circles.

**Figure A2:**
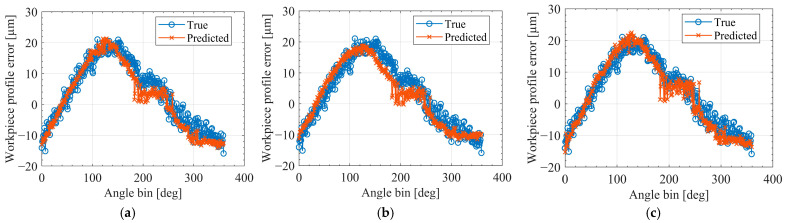
Predicted and measured workpiece profile error for one typical run using XGBoost (**a**), MLP (**b**), and RF (**c**).

## Data Availability

The raw data supporting the conclusions of this article will be made available by the authors on request.

## Appendix A. GUM-Consistent Traceability and Type-A Uncertainty of Ballbar Measurements

Ballbar measurements were conducted off-line prior to machining for five times. The ballbar length signals were mapped to a common angular grid $\theta_{k}$∈[0°, 360°], rigidly registered to remove low-order eccentricity and tilt by using least-square fitting method, then projected to obtain the point-wise radial error (Figure A1a). This $e_{r}\left(\theta \right)$ serves as the geometric-anchor feature.

Under the same setting, *n* = 5 repeated CCW circles were acquired. For the *i*-th circle, define the ring RMS

(A1) $${RMS}_{i}=\sqrt{\frac{1}{K}\sum_{k=1}^{K}{\left(e_{r}^{\left(i\right)}(\theta_{k})\right)}^{2}}$$

where K=360 is the number of sampled angular nodes per revolution. The calculated RMS of the five measurements could be found at Figure A1b. Let s be the across-run sample standard deviation of ${RMS}_{i}$. The ring-level Type-A standard uncertainty is

(A2) $$u_{A}=\frac{s}{\sqrt{n}}$$

Finally, the ring-level Type-A equals 0.17 μm, indicating sub-micrometer repeatability for the off-line ballbar labels in the machining direction.

In the terminology of GUM, this Type-A standard uncertainty $u_{A}$ quantifies only the random repeatability component associated with the ballbar input quantity. Other input quantities that influence the overall measurand y(θ)—the pointwise radial deviation of the machined circular profile referenced to the CMM baseline—include the CMM radius measurement, time-to-angle synchronization, interpolation and resampling of the ballbar signal, accelerometer calibration and noise, thermal drift within a run, alignment between the ballbar and CMM frames, and residual modeling error. Reliable calibration certificates and detailed environmental data for these components are not yet available for the present test bench. A complete numerical GUM-consistent evaluation of the combined standard uncertainty $u_{c}$ and the expanded uncertainty U is therefore outside the scope of this study. We restrict ourselves to reporting the ballbar Type-A component $u_{A}$ and we identify the construction of a full error budget as an important topic for future work.

## Appendix B. Hyperparameter Optimization

The main text provides only an overview of the methods and tuning strategies. Detailed model hyperparameters and their corresponding search ranges are presented in Table A1 to support reproducibility and facilitate future extensions.

**Table A1:** Hyperparameter Search Space for Model Tuning (Python Syntax).

Methods	Parameters	Value
XGBoost	n_estimators	100, 200, 300, 400, 500
	max_depth	3, 5, 7, 9
	learning_rate	0.01, 0.05, 0.1, 0.2
	subsample	0.6, 0.8, 1.0
	colsample_bytree	0.6, 0.8, 1.0
	gamma	0, 1, 5
	reg_lambda	0.1, 1.0, 10.0
	early_stopping_rounds	10
MLP	learning_rate	0.0005, 0.001, 0.005, 0.01
	reg_lambda	1 × 10^−5^, 1 × 10^−4^, 1 × 10^−3^
	hidden_layer_sizes	(64,64), (128,64), (128,128), (256,128)}
	activation	‘relu’, ‘tanh’
	solver	‘adam’
	max_iter	300
RF	n_estimators	100, 200, 300, 400, 500
	max_depth	5, 10, 15, 20
	min_samples_split	2, 4, 6, 8
	min_samples_leaf	1, 2, 4
	max_features	‘auto’, ‘sqrt’, ‘log2’

## Appendix C. Meaning of Features for AI Model

This appendix provides a detailed description of the input features used in the machine learning models. As outlined in Section 4.2, all features are statistical descriptors derived from tri-axial vibration signals along the X, Y, and Z axes, as well as from the pre-machining circular path error measured by the ballbar device. For each acceleration channel, five statistics are computed in the angular domain: Mean, SD, RMS, P2P, and Cf. These descriptors capture distinct aspects of the vibration signal, characterizing the Mean, SD, RMS, P2P, and Cf of the machining process. The ballbar-derived path error serves as a static geometric reference along the circular trajectory.

To support the ablation experiments presented in Section 6.3, the features are organized into six nested configurations: F-16: The full feature set, comprising the ballbar path error and all five statistical descriptors from each of the three acceleration axes, totaling sixteen variables; F15: A vibration-only set, which excludes the path error and retains all fifteen acceleration-based features. This configuration isolates the predictive contribution of inertial signals; F7-A: A compact hybrid set combining the path error with low-order vibration descriptors (Mean and SD from the X, Y, and Z axes), emphasizing overall vibration level and variability; F7-B: An alternative hybrid set combining the path error with high-amplitude descriptors (P2P and Cf from the three axes), targeting dynamic range and impulsive events; F6-A: A vibration-only subset consisting of Mean and SD from the three axes; the ballbar feature is excluded; F-6-B: A complementary vibration-only subset containing P2P and Cf from the three axes, also excluding the path error.

Table A2 summarizes these feature groups, including the detailed definitions, physical interpretations, and axis of origin for each descriptor.

**Table A2:** Features for AI model development.

Feature Type	Feature Explanation
F16	Acc of X-axis (X-Mean; X-SD; X-RMS; X-P2P; X-Cf) Acc of Y-axis (Y-Mean; Y-SD; Y-RMS; Y-P2P; Y-Cf) Acc of Z-axis (Z-Mean; Z-SD; Z-RMS; Z-P2P; Z-Cf) Path error
F15	Acc of X-axis (X-Mean; X-SD; X-RMS; X-P2P; X-Cf) Acc of Y-axis (Y-Mean; Y-SD; Y-RMS; Y-P2P; Y-Cf) Acc of Z-axis (Z-Mean; Z-SD; Z-RMS; Z-P2P; Z-Cf)
F7-A	Acc of X-axis (X-Mean; X-SD) Acc of Y-axis (Y-Mean; Y-SD) Acc of Z-axis (Z-Mean; Z-SD) Path error
F7-B	Acc of X-axis (X-P2P; X-Cf) Acc of Y-axis (Y-P2P; Y-Cf) Acc of Z-axis (Z-P2P; Z-Cf) Path error
F6-A	Acc of X-axis (X-Mean; X-SD) Acc of Y-axis (Y-Mean; Y-SD) Acc of Z-axis (Z-Mean; Z-SD)
F6-B	Acc of X-axis (X-P2P; X-Cf) Acc of Y-axis (Y-P2P; Y-Cf) Acc of Z-axis (Z-P2P; Z-Cf)

## Appendix D. Predicted Workpiece Profile Error Using XGBoost, MLP and RF

As shown in Figure A2, all three regressors are able to replicate the general shape of the error curve. However, XGBoost exhibits closer alignment with the measured profile, particularly in its ability to track both the rising and falling flanks with reduced local deviations compared to the multilayer perceptron (MLP) and random forest (RF), especially in the vicinity of the peak region. This visual comparison is consistent with the residual statistics and coverage indicators reported previously, and it illustrates how differences in global performance metrics manifest in the pointwise prediction behavior along the circular trajectory.

In addition to comparing different regressors, it is instructive to examine the impact of input feature selection on the pointwise predictions of XGBoost. Figure A3 presents the predicted and measured workpiece profile errors for a representative run using the F15, F7-A, F7-B, F6-A, and F6-B feature configurations. While all hybrid sets preserve the general shape of the error curve, the compact F7-A and F7-B configurations yield predictions that closely follow the measured profile, exhibiting only minor local deviations. In contrast, the vibration-only sets F6-A and F6-B demonstrate notably larger discrepancies, particularly near the peak region and along the descending flank. These observations are consistent with the quantitative ablation results and further substantiate the role of ballbar-derived features as critical geometric references for achieving high-fidelity trajectory-level predictions.

## References

[1] Ji W., Wang L. (2019). Industrial robotic machining: A review. Int. J. Adv. Manuf. Technol..

[2] Kim S.H., Nam E., Ha T.I., Hwang S.-H., Lee J.H., Park S.-H., Min B.-K. (2019). Robotic Machining: A Review of Recent Progress. Int. J. Precis. Eng. Manuf..

[3] Schmitz T.L., Couey J., Marsh E., Mauntler N., Hughes D. (2007). Runout effects in milling: Surface finish, surface location error, and stability. Int. J. Mach. Tool Manufact..

[4] Navarro-Devia J.H., Chen Y., Dao D.V., Li H. (2023). Chatter detection in milling processes—A review on signal processing and condition classification. Int. J. Adv. Manuf. Technol..

[5] Petráček P., Fojtů P., Kozlok T., Sulitka M. (2022). Effect of CNC Interpolator Parameter Settings on Toolpath Precision and Quality in Corner Neighborhoods. Appl. Sci..

[6] Wang Z., Zhang R., Keogh P. (2020). Real-Time Laser Tracker Compensation of Robotic Drilling and Machining. J. Manuf. Mater. Process..

[7] Archenti A., Nicolescu M., Casterman G., Hjelm S. (2012). A New Method for Circular Testing of Machine Tools Under Loaded Condition. Procedia CIRP.

[8] Li X., Liu H. (2008). Measurement and analysis of typical motion error traces from a circular test. Front. Mech. Eng. China.

[9] Wuyang S.U.N., Dinghua Z., Ming L.U.O. (2021). Machining process monitoring and application: A review. J. Adv. Manuf. Sci. Technol..

[10] Kim H., Nam S., Nam E. (2023). Estimation of Shape Error with Monitoring Signals. Sensors.

[11] Fu X., Song H., Li S., Lu Y. (2025). Digital twin technology in modern machining: A comprehensive review of research on machining errors. J. Manuf. Syst..

[12] Fu X., Li S., Song H., Lu Y. (2025). Digital Twin-driven multi-scale characterization of machining quality: Current status, challenges, and future perspectives. Robot Comput. Integrated Manuf..

[13] Nan J., Yu S., Liu C., Chen M., Sun Y. (2025). Online compensation of contour errors in robotic milling based on multi-source characterization and path reconstruction. Chin. J. Aeronaut..

[14] Li R., Ding N., Zhao Y., Liu H. (2023). Real-time trajectory position error compensation technology of industrial robot. Measurement.

[15] Bilancia P., Ferrarini S., Berni R., Pellicciari M. (2024). Assessing path accuracy in industrial robots via ballbar technology. Ind. Rob..

[16] Li Z., Dai Y., Guan C., Lai T., Sun Z., Li H. (2024). An on-machine measurement technique with sub-micron accuracy on a low-precision grinding machine tool. J. Manuf. Process..

[17] Yao Z., Zhang P., Luo M. (2024). Extreme learning machine oriented surface roughness prediction at continuous cutting positions based on monitored acceleration. Mech. Syst. Signal Process..

[18] Jones T., Cao Y. (2025). Tool wear prediction based on multisensor data fusion and machine learning. Int. J. Adv. Manuf. Technol..

[19] Chen J., Lin J., Zhang M., Lin Q. (2024). Predicting Surface Roughness in Turning Complex-Structured Workpieces Using Vibration-Signal-Based Gaussian Process Regression. Sensors.

[20] Chen B., Zha J., Cai Z., Wu M. (2025). Predictive modelling of surface roughness in precision grinding based on hybrid algorithm. CIRP Ann. Manuf. Sci. Technol..

[21] Huang P.-M., Lee C.-H. (2021). Estimation of Tool Wear and Surface Roughness Development Using Deep Learning and Sensors Fusion. Sensors.

[22] Plaza E.G., López P.J.N., González E.M.B. (2018). Multi-Sensor Data Fusion for Real-Time Surface Quality Control in Automated Machining Systems. Sensors.

[23] Wang J., Wu X., Huang Q., Mu Q., Yang W., Yang H., Li Z. (2025). Surface roughness prediction based on fusion of dynamic-static data. Measurement.

[24] Klimchik A., Furet B., Caro S., Pashkevich A. (2015). Identification of the manipulator stiffness model parameters in industrial environment. Mech. Mach. Theory.

[25] Díaz-Tena E., Ugalde U., de Lacalle L.N.L., de la Iglesia A., Calleja A., Campa F.J. (2013). Propagation of assembly errors in multitasking machines by the homogenous matrix method. Int. J. Adv. Manuf. Technol..

[26] Bustillo A., Urbikain G., Perez J.M., Pereira O.M., de Lacalle L.N.L. (2018). Smart optimization of a friction-drilling process based on boosting ensembles. J. Manuf. Syst..

[27] Zhou J., Liu X., Liao Q., Wang T., Wang L., Yang P. (2025). Multi-Sensor Heterogeneous Signal Fusion Transformer for Tool Wear Prediction. Sensors.

[28] Wu M., Yao Z., Verbeke M., Karsmakers P., Gorissen B., Reynaerts D. (2025). Data-driven models with physical interpretability for real-time cavity profile prediction in electrochemical machining processes. Eng. Appl. Artif. Intell..

[29] Yao M., Chen Z., Li J., Guan S., Tang Y. (2025). Ultrasonic identification of CFST debonding via A novel Bayesian Optimized-LSTM network. Mech. Syst. Signal Process..

[30] Chen S., Ellwein C., Klingel L., Neumann R., Zhang J., Riedel O., Verl A., Wortmann A. (2025). Digital twins for machine tools: A systematic mapping study. Digit. Twin.

[31] Xing K., Bonev I.A., Liu Z., Champliaud H. (2024). Influence of machining parameters on dynamic errors in a hexapod machining cell. Int. J. Adv. Manuf. Technol..

[32] Shu T., Gharaaty S., Xie W., Joubair A., Bonev I.A. (2018). Dynamic Path Tracking of Industrial Robots with High Accuracy Using Photogrammetry Sensor. IEEE/ASME Trans. Mechatron..

[33] Wang Z., Mastrogiacomo L., Franceschini F., Maropoulos P. (2011). Experimental comparison of dynamic tracking performance of iGPS and laser tracker. Int. J. Adv. Manuf. Technol..

[34] Arnaiz-González Á., Fernández-Valdivielso A., Bustillo A., López de Lacalle L.N. (2016). Using artificial neural networks for the prediction of dimensional error on inclined surfaces manufactured by ball-end milling. Int. J. Adv. Manuf. Technol..

[35] Xing K., Cianyi Y., Bonev I., Champliaud H., Liu Z. (2025). Offline Circular Path Error Measurement and Compensation for Robotic Machining Applications. Meas. Sci. Technol..

[36] Ma L., Howard I., Pang M., Wang Z., Su J. (2020). Experimental Investigation of Cutting Vibration during Micro-End-Milling of the Straight Groove. Micromachines.

[37] Xing K., Bonev I.A., Liu Z., Champliaud H. (2024). Positioning performance of a hexapod machining cell under machining and nonmachining operations. J. Mech. Sci. Tech..

[38] Chen T., Guestrin C. XGBoost: A Scalable Tree Boosting System. Proceedings of the 22nd ACM SIGKDD International Conference on Knowledge Discovery and Data Mining.

[39] Amjad M., Ahmad I., Ahmad M., Wróblewski P., Kamiński P., Amjad U. (2022). Prediction of Pile Bearing Capacity Using XGBoost Algorithm: Modeling and Performance Evaluation. Appl. Sci..

[40] Bentéjac C., Csörgő A., Martínez-Muñoz G. (2019). A Comparative Analysis of XGBoost. Artif. Intell. Rev..

[41] Wan S., Li S., Chen Z., Tang Y. (2025). An ultrasonic-AI hybrid approach for predicting void defects in concrete-filled steel tubes via enhanced XGBoost with Bayesian optimization. Case Stud. Constr. Mater..

[42] Schmidhuber J. (2015). Deep learning in neural networks: An overview. Neural Netw..

[43] Bengio Y., Montavon G., Orr G.B., Müller K.-R. (2012). Practical Recommendations for Gradient-Based Training of Deep Architectures. Neural Netw. Tricks Trade.

[44] Montavon G., Samek W., Müller K.-R. (2018). Methods for interpreting and understanding deep neural networks. Digit. Signal Process..

[45] Breiman L. (2001). Random Forests. Mach. Learn..

[46] Liaw A., Wiener M. (2001). Classification and Regression by RandomForest. Forest.

[47] Probst P., Wright M., Boulesteix A.-L. (2019). Hyperparameters and tuning strategies for random forest. WIREs Data Min. Knowl. Discov..

[48] Tarwidi D., Pudjaprasetya S.R., Adytia D., Apri M. (2023). An optimized XGBoost-based machine learning method for predicting wave run-up on a sloping beach. MethodsX.

[49] Bergstra J., Bengio Y. (2012). Random search for hyper-parameter optimization. J. Mach. Learn. Res..

[50] Tatachar A.V. (2021). Comparative assessment of regression models based on model evaluation metrics. Int. Res. J. Eng. Technol. (IRJET).

[51] Strobl C., Boulesteix A.-L., Zeileis A., Hothorn T. (2007). Bias in random forest variable importance measures: Illustrations, sources and a solution. BMC Bioinform..

[52] Ge J., Yao Z., Wu M., Almeida J.H.S., Jin Y., Sun D. (2025). Tackling data scarcity in machine learning-based CFRP drilling performance prediction through a broad learning system with virtual sample generation (BLS-VSG). Compos. Part B Eng..

